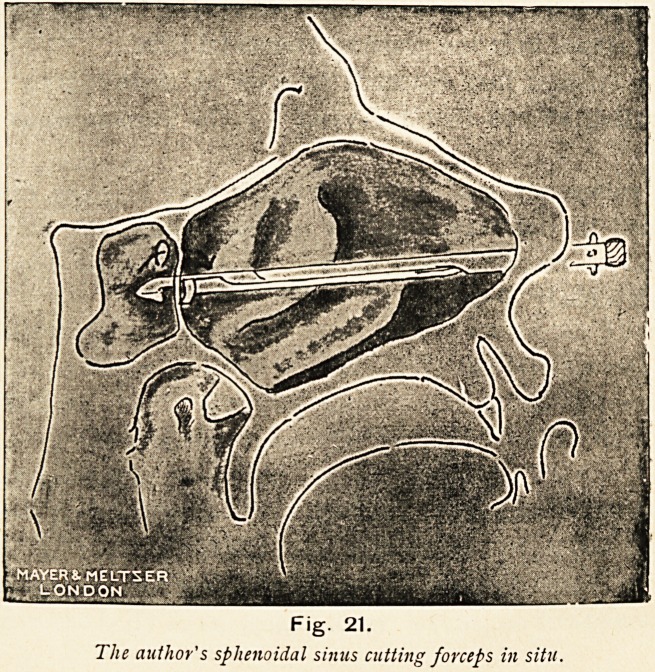# The Long Fox Lecture: Suppurative Disease in the Nose and Ear

**Published:** 1908-03

**Authors:** Patrick Watson Williams

**Affiliations:** Lecturer on Diseases of the Nose and Throat, University College, Bristol; Laryngologist and Rhinologist, Bristol Royal Infirmary. Consulting Surgeon for Diseases of the Ear and Throat, Pontypool Hospital


*" Scire est nescire, nisi id me*
*Scire alius sciret."*

MARCH, 1908.

### THE LONG FOX LECTURE:

THE FOURTH ANNUAL LECTURE ARRANGED BY THE COMMITTEE OF THE LONG FOX MEMORIAL,
DELIVERED IN THE MEDICAL LIBRARY, UNIVERSITY COLLEGE, BRISTOL,
ON JANUARY 16TH, 1908.

J. MICHELL CLARKE, M.D., F.R.C.P., *in the Chair.*

BY

PATRICK WATSON WILLIAMS, M.D. Lond.,
*Lecturer on Diseases of the Nose and Throat, University College, Bristol;*
*Laryngologist and Rhinologist, Bristol Royal Infirmary.*
*Consulting Surgeon for Diseases of the Ear and Throat, Pontypool Hospital.*

ON

### SUPPURATIVE DISEASE IN THE NOSE AND EAR, WITH SPECIAL REFERENCE TO SOME NEWER METHODS OF TREATMENT.

---

THESE lectures were instituted to serve a two-fold purpose: firstly, to keep alive the memory of a physician who as a teacher of the art and science of medicine laboured in this Bristol Medical School long and well, whose illustrious example taught not alone what medical science had brought to the service of man, but what the type of a true physician should be; and, secondly, to encourage and promote original thought and investigation in the many phases of what is comprised in the now comprehensive term science.

To be appointed one of those to weave this garland around the memory of Edward Long Fox is a privilege of which I am justly proud, and not less so because I am convinced that on this occasion it is a personal connection, rather than scientific merit, that has led to my being asked to add one laurel to the wreath.

More distinguished predecessors in this lectureship have expressed the high regard in which Long Fox was held by his medical contemporaries, but no one could have been in a better position than myself to feel the inspiration of his personality, or to realise the simplicity, deep sincerity, and warm, sympathetic nature that was here combined with perennial geniality and such a keen sense of humour. He did not scorn delights, though he lived laborious days. "Of every friendless name the friend," he combined a love for his work with a deep love for all men, and to the last devoted much time and work in the poorest parish in the city. His innate good sense and enviable power of expressing real sympathy made him not only widely loved in his professional as well as in his family life, but one to whom all instinctively turned in times of difficulty or sorrow.

> "As with us mortal men, the laden heart
> Is persecuted more, and fevered more,
> When it is nighing to the mournful house
> Where other hearts are sick of the same bruise."

He lived a simple life, generous to everyone but himself; cultured and scholarly, he instinctively abhorred anything in the nature of show. Of Fox it might truly be said—

> "When fainting nature called for aid,
> And hovering death prepared the blow,
> His vigorous remedy displayed
> The power of art without the show."

In no one department of medicine or surgery has there been greater and more rapid development of our knowledge of diseased processes and methods of treatment than in diseases of the nose and ear, and though only one phase of diseases in this restricted field comes within the scope of this lecture, it is scarcely possible to enter fully into the symptoms and treatment, much less the many diagnostic methods employed in practice.

It is more particularly the comparatively new field of nasal accessory purulent discharges that I propose to discuss, dwelling especially on some features which are directly interesting in general practice, and on some newer methods of treatment, more particularly those methods which I have found most effective.

Before turning to other questions, it may be useful to recall some of the more important points in the clinical anatomy of the regions to be discussed.

Fig. 1.

*Diagram showing the arrangement of the nasal accessory sinuses, and their division into an anterior and posterior group by the line of insertion of the middle turbinated body.*

For clinical purposes it is convenient to divide the accessory sinuses into two groups, viz. :—

(1) The anterior group, comprising those which open into the middle meatus, *i.e.* in front of and below the attachment of the middle turbinated body, viz. the frontal sinus and anterior (and middle) ethmoid cells, and the maxillary antrum.

(2) The posterior group, comprising those which open into the superior meatus, *i.e.* behind and above the attachment of the middle turbinal, viz. the posterior ethmoid cells and the sphenoidal sinus.

Thus not only anatomically, but also clinically the ethmoid cells occupy a central and connecting relationship to the other nasal accessory sinuses. The ethmoid labyrinth is complicated by the frequency with which it develops irregularly, so as to encroach on these other cavities, and in the diagnosis of sinus suppuration it is essential that their anatomical arrangement should be understood.

The position of the labyrinth in the nasal cavity, and its relation to the orbit, the maxillary antrum, and the floor of the anterior cranial fossa, is well shown in the transverse section of the skull (Fig. 2).

Fig. 2.

*Frontal section showing right side of nose. Cross section through ethmoidal labyrinth* (SHAMBAUGH).

If now we examine a sagittal section (Fig. 3), in which the middle turbinated body has been partially detached, we observe certain fairly constant partition plates which form prominences projecting towards the nasal passage, viz. :—

1. The plate of the uncinate process.
2. The plate of the bulla ethmoidalis.
3. The plate of the middle turbinated body (concha media).
4. The plate of the superior turbinated body (concha superior).
5. The plate of the fourth turbinated body (concha suprema). This is very frequently rudimentary.

Fig. 3.

*Sagittal section showing left side. Preparation shows typical construction of ethmoid labyrinth; the several partition plates presenting an unusually simple form* (SHAMBAUGH).

Fig. 4.

*Sagittal section showing left side. The preparation shows an ethmoid cell has been developed below the unciform plate, pushing this up into the frontal sinus, where a bulla frontalis is formed; the other plates of the ethmoid are formed as usual.* (SHAMBAUGH).

Fig. 5.

*Transverse section showing a large middle turbinal cell* (ROE).

Between these prominences are grooves in which the various cells and sinuses open; thus the groove between the uncinate process and ethmoid bulla is called the infundibulum, and the mouth of this groove is called the hiatus semilunaris.

Into this groove open the maxillary antrum, anterior or infundibular ethmoid cells, and, as a rule, the anterior end of the groove opens into the frontal sinus; often, however, the groove ends blindly, dilating into an ethmoid cell which projects into the floor of the frontal sinus, and is then termed a bulla frontalis.

Between the second and third plates is another groove, the groove of the bulla, into which open the rest of the anterior, or as they are sometimes called, the middle ethmoid cells.

Between the third and fourth plates, *i.e.* in the superior meatus the posterior ethmoid cells open, and the sphenoidal sinus opens more posteriorly into this groove.

Sometimes a large ethmoid cell develops in the unciform process, forming a prominent agger nasi; again, a middle or

Fig. 6.

*Diagrams showing various developmental irregularities of the frontal sinuses and their relations to the roof of the nasal passages.*

posterior ethmoid cell or cells may develop forwards so as to encroach on the frontal sinus, or come to lie between the orbital roof and the floor of the anterior cranial fossa (supra-orbital ethmoidal cells); or, again, an ethmoid cell may develop backwards so as to lie over the sphenoidal sinus, giving the condition described as double sphenoidal sinus. Again, by the development of a large ethmoid cell in the anterior end of the middle turbinated body a cystic enlargement may be produced there, known as a concha bullosa.

Thus great irregularity of the ethmoid cells arises, such irregular development being at the expense of the other sinuses, and is liable to cause difficulties in diagnosis and in operative treatment.

It is mainly due to the irregular development of the frontal sinuses, and their relationship to the ethmoidal cells, that difficulty arises in the course of operation.

It will be seen (*a*) that pus discharging from any of the anterior group will appear in the middle meatus *beneath the middle turbinated body,* and that it will tend to pass towards the anterior nasal orifice, especially on stooping forwards. Again (*b*), pus coming from any of the posterior group must appear above the middle turbinal, that is, in the olfactory fissure: further, it enters the nasal passage far back, and will tend to pass backwards into the naso-pharynx, being guided in this direction by the shape and direction of the middle turbinated body. Hence pus coming from this group, the sphenoidal sinus and posterior ethmoidal cells, can often be observed in the choanæ, by posterior rhinoscopy, appearing above the posterior end of the middle turbinated body.

Fig. 7.

*Frontal section through the right orbit, showing frontal sinus and ethmoid cells which have developed out over the orbit. The latter if suppurating would complicate an operation for frontal sinusitis unless specially sought for and opened up* (SHAMBAUGH)

Reference to anatomical preparations further makes it clear that empyema of the sphenoidal sinus and posterior ethmoidal cells must tend to be associated with one or more of the neighbouring cavities So, too, it is inevitable that if the frontal sinus is secreting pus the anterior ethmoidal cells must very usually become involved, while pus from any of these upper cells, opening as they do into the infundibulum and hiatus semilunaris, often in part enters the opening of the maxillary antrum, causing secondary empyema there.

Again, from the relative position of the various cavities and their opening into the nasal passages, we see it follows that if a maxillary antrum contains pus, so long as the patient stands upright, it will not overflow into the nose till the pus rises up to the level of the opening in the middle meatus, and then will

Fig. 8.

*Section of the left temporal, showing the tympanum from the inside, the iter ad antrum and the mastoid antrum and cells. In front, i.e. to the right, is seen the canal for the Eustachian tube.*

constantly discharge ; but on stooping low or turning the head to the opposite side, the pus will flow freely from the full antrum, so that patients complain of pus dripping from the nose on stooping or running on to the pillow at night if they lie on one side.

Moreover, the antrum can hardly empty itself ; consequently the pus gets infected with saprophytic organisms and stinks, causing a subjective foul odour or taste, cacosmia or cacogeusia.

As the frontal sinus opening is the lowest point of the cavity, the pus tends to collect during the night when the patient is lying down ; but provided the outlet is not closed, it begins running out soon after rising, and continues running freely for an hour or two till the cavity has emptied, and then it comes away gradually in small quantities.

In pure ethmodial suppuration the pus is constantly running, but generally in small amount; although when the ethmoid cells are over-developed, it will be realised that the secretion may be as copious as from any of the larger sinuses.

In these nasal accessory sinuses, as in the mastoid antrum, connected as it is with the tympanic cavity through the *iter ad antrum*, we have one feature common to all alike, they are bony cavities lined with mucous membrane, all potentially open and exposed to infection from the respiratory tract, all having small apertures of exit tending to cause retention of infective secretions with consequent formation of pus. The symptoms arising in every case depend essentially on such retention, and while owing to variations in anatomical relationship the signs and symptoms differ, the same essential surgical principle applies to the methods adopted for their cure, viz. free drainage.

### SYMPTOMS OF CHRONIC SUPPURATION IN THE ACCESSORY SINUSES.

In drawing attention to some of the more important symptoms, it is convenient and instructive to group the symptoms from the clinical standpoint, rather than the anatomical, under the following heads :—

(*a*) **Nervous symptoms.** *Neuralgia and headache.*—The most constant symptom is periodic headache, due to accumulation of secretion in a cavity causing pressure and irritation of the sensory nerves, until the increasing pressure forces an exit for the purulent mucus through the natural ostium, narrowed and closed by the swelling of the lining mucosa: relief following the escape of pus, until with its re-accumulation the headache returns and the process is repeated. The commonest seat of pain is the supraorbital region, the vertex or the occiput, and often it does not correspond to the particular cavity involved, being a referred pain. Thus antral empyema very usually causes headache in any one or all of these regions, and I would particularly emphasise the fact that simple occipital headache may be due to antral disease.

Neuralgia due to frontal sinus disease is usually supra-orbital, and often there is marked tenderness on deep pressure at the upper internal angle of the orbital roof. The symptoms in their character and periodicity, particularly in unilateral sinusitis, are often suggestive of migraine, and in many patients an erroneous diagnosis of migraine has been made until the import of an associated nasal discharge came to be recognised.

In sphenoidal sinus empyema the pain is often deep and very severe, and yet its situation difficult to describe, the headache being accompanied by a marked feeling of mental confusion, and sometimes patients say they feel as if they must go out of their mind. But there is one symptom which is very misleading—pain in the ear. It is not often present, even in sphenoidal sinus disease ; but if it is the only pain the patient experiences, the seat of trouble is liable to be completely overlooked.

Last summer I was asked by Dr. H. Willcox to see a lad aged 12, in consultation, for acute pain in the right ear, with slight febrile disturbance and photophobia. It had lasted a day or two, and the symptoms suggested acute middle ear disease. But the membrana was normal, and there was entire absence of any tenderness over the mastoid, or other indications of aural trouble. On examining the nose, there was some slight rhinitis, and the previous history seemed to point to a mild influenza attack. I came to the conclusion that he had recent sphenoidal sinusitis and gave a very guarded prognosis, notwithstanding the absence of alarming symptoms, for the boy had not felt ill enough to be in bed.

We decided on a course of local inhalations, sedative sprays containing cocaine and suprenine, with the hope of causing the sinus to evacuate itself, his condition at that time not being sufficiently urgent to warrant opening the sphenoidal sinus, a procedure which is very difficult and dangerous in so young a child with undeveloped sphenoidal sinuses. Not improving, he was seen by Dr. Arthur Cheatle, who decided that, at any rate, there was nothing calling for aural operation. After a further interval, when the headache had become general, and the child was only semi-conscious, Sir Victor Horsley saw him, and then diagnosed "influenzal cerebrospinal meningitis." At the *post-mortem* examination an accumulation of pus was found around the right cavernous sinus. There is good reason to believe that influenza infected the sphenoidal sinus, causing sphenoidal sinusitis and pain in the ear, and that the infection spread through the lymphatic channels and the subarachnoid space surrounding the cavernous sinus, and hence spread throughout the subarachnoid space.

Tilley had a case of sphenoidal sinus disease in which severe pain in the ear had led a surgeon to advise a radical mastoid operation. He was able to reproduce the intense aural pain by pressure in the sphenoidal sinus with a cotton wool probe. Two other cases of sphenoidal sinus suppuration with severe pain in the ear are referred to by St. Clair Thomson, and in both competent surgeons were induced to open the mastoid to no purpose.

All patients suffering from purulent sinusitis are liable to suffer from general toxæmic symptoms, toxic products reaching the blood either from direct absorption from the implicated sinuses or from the gastro-intestinal tract. But lymphatic absorption of toxic matters, in the presence of channels of communication with the intracranial venous sinuses, must be held to account for the profound mental depression and difficulty in thinking clearly, almost amounting to slow cerebration, that is very frequently present in fronto-ethmoidal or spheno-ethmoidal sinus suppuration, and sometimes, to a less marked degree, even in simple maxillary sinus disease. Many patients have come with drawn, sad expression and sallow complexion, expressing their weariness of life and a profound melancholia which was quite foreign to their natural state of mind, symptoms which have completely disappeared with the removal of their sinus disease. Sometimes it has been difficult to recognise in the round-faced, cheerful individual one who shortly before treatment was a haggard melancholic.

I have never met with a case where definite mental aberration preceded and was relieved by accessory sinus treatment, but they are by no means unknown. Stucky[1] relates a series of cases coming under his own observation where definite mental symptoms and suicidal tendencies in many of them were completely cured by operations on the sinuses.

From extension of sphenoidal sinus infection especially, but also from frontal and ethmoidal suppurations, such unfortunate

[1] *Med. Rec.*, 1906, lxx. 820.

complications as cavernous sinus and petrosal sinus thrombosis, subdural and intradural abscess, suppurative meningitis, brain abscess, erosion of the carotid and other endo-cranial vessels, have been known to arise, and Schech reports a case in which in addition there was glycosuria with polyuria.

Fig. 9.

*One of the author's cases of sphenoidal sinus suppuration, with cavernous sinus thrombosis and consequent exophthalmos.*

*Paroxysmal sneezing, rhinorrhœa and asthma,* are among the other more frequent neuroses set up by accessory sinus disease.

I will refer to the first-mentioned, paroxysmal sneezing and rhinorrhœa, only to emphasise the fact that in the earlier stages of ethmoidal suppuration, when there may be nothing to observe on examination but a tumid redness and a fulness of the middle turbinal body, these symptoms, which are generally pure neuroses, may be really due to a chronic inflammatory infective lesion, which if unattended to will eventuate in the formation of polypi, and not unlikely with extension of the suppurative process to the other sinuses. I venture to touch on the relationship of spasmodic asthma to nasal suppuration, although it is too large a question to deal with at all fully. But I have had such conclusive evidence in cases under my own observation that true spasmodic asthma may be due to suppurative nasal disease, as to leave me in no doubt of their interdependence. Since the classical case recorded by Voltolini, where the removal of a nasal polypus cured long-standing asthma, the question of nasal polypus being a cause of asthma has long been a hotly-debated subject. The very large percentage of patients with large nasal polypi who do not suffer from asthma, and the very large percentage of true asthmatics in whom no nasal polypi can be found, tend to prove fairly conclusively that there is no direct connection between nasal polypus and asthma as cause and effect. The nasal abnormalities which are frequently associated with asthma are œdematous swelling on the middle turbinals, a general œdematous infiltration of the Schneiderian membrane, septal deformities causing more or less obstruction, and in young children adenoid growths. Such conditions are causes of intra-nasal excitation, and similar conditions result from infective inflammations in the accessory cavities, which, while eventuating in many cases in the formation of polypi, are also efficient peripheral causes of asthma in those with unstable nerve centres; afferent impulses from these are as influencing the bulbar respiratory centres, and through them the efferent nerves to the bronchioles.

Bronchial asthma is probably an exaggeration of bronchiolar contraction and dilation in expiration and inspiration, which probably occurs normally, just as the alæ nasi and the glottic opening dilate with deep inspiration to contract with expiration. Evidence in support of this I have discussed at length elsewhere,[1] and I may point out that Cajal has demonstrated in the bulb of a mouse that a few of the collateral fibres from the gelatinous substance of Rolando (the receptive nucleus of the fifth nerve) break up under the motor nuclei of the facial and vagus, and the inference is that they communicate. That being the case, we have an explanation of

[1] Watson Williams.

the influence of the sensory areas of the fifth nerve, especially of the nasal mucosa, on certain regions in the motor territory of the vagus.

(*b*) **Ocular Symptoms.**—That displacement of an eye, with consequent strabismus, may result from distension of an accessory sinus in closed empyema has long been a matter of common knowledge. Thus a frontal sinus empyema or mucocele may displace the eye downwards and outwards; distension of ethmoid cells with protrusion of the orbital plate displaces the eye outwards. These gross displacements of the eye are rare, but many other ocular symptoms are much more frequently attributable to sinus suppuration than is generally recognised. With either antral empyema, frontal or sphenoidal sinus suppuration, blurred vision, a tendency to lachrymation and conjunctival injection of the corresponding eye are often noticed by patients on reading or doing fine work. A moderate degree of œdema of the eyelids is frequently seen even in early cases of fronto-ethmoidal sinusitis, especially in the upper eyelid and on the nasal side. These are probably due to vaso-motor disturbances from irritation of the sympathetic branches connected with the ganglia associated with the fifth nerve. Posey[1] draws attention to the non-inflammatory nature of this simple œdema, which is to be distinguished from inflammatory thickening of the lid in cellulitis. Another class of cases described by Posey are those designated "pre-lachrymal abscess," the term by which he refers to the swelling which sometimes forms above the internal palpebral ligament, and somewhat external to the lachrymal sac. These pre-lachrymal abscesses, which are sometimes mistaken for abscess of the sac itself, are often due to necrosis of the lachrymo-ethmoidal cells, or to a frontal sinus suppuration pointing here.

Again, moderate degrees of neuritis, Posey finds, may frequently be diagnosed by the distension of the lymph sheaths of the retinal vessels, and objectively by a diminution of a light sense, and a sense of fulness in the eye, with pain on rotation.

Dragging pain at the back of the eyeball is a usual symptom in sphenoidal sinus disease. St. Clair Thomson, who collected

[1] *Med. Rec.*, 1907, lxxii. 255.

forty-two cases of sphenoidal sinus suppuration with cerebral and ophthalmic complications, considers that "perhaps the only pain which is characteristic of sphenoidal sinus disease is when the patient states that it is deep in behind the eyes. It may be so intense as to cause insomnia."

Fig. 10.

*Frozen section of the sphenoidal sinuses and its anatomical relations* (HOLMES).

It is not until the intra-cranial extension of the infection and inflammation supervenes that the various nerves in and around the cavernous sinus, and in close proximity to the roof or below the sinus, give definite indications of being involved.

From the position of the optic chiasma and nerves, the nerves passing through the cavernous sinus, and the close proximity of the superior maxillary nerve and of its branches to Meckel's ganglion, we can readily understand that congestion of the retinal veins, papillary stasis, œdema, atrophy and chemosis, paralysis of the ocular muscles, with consequent ptosis, strasbimus, unilateral and bilateral temporal hemianopsia, and eventually cavernous sinus phlebitis and thrombosis or abscess, and exophthalmos are liable to ensue. Vision may be good or visual fields contracted, or complete amaurosis may arise gradually or even suddenly, from thrombosis of the retinal vein. Panophthalmitis and destruction of the eyeball has been observed; but apart from such unfortunate complications, the failure to recognise the interdependence of the ocular manifestations to accessory sinus disease has led to unnecessary enucleations of the eyeball. Fish[1] refers to several such instances coming under his notice.

It is remarkable, however, that definite external muscle paralysis may arise without other ocular symptoms. Thus Bryan[2] relates a case of sphenoidal sinus disease in which there was paralysis of both external recti but no other changes, and no pain in the eye. I have spoken of these graver ocular lesions as being due to sphenoidal sinus disease, but they may one and all equally be caused by posterior ethmoidal cell suppuration, for Onodi has shown that these cells are not seldom in direct relation with the cavernous sinus and the optic nerve or optic chiasma.

It is worthy of note too, as demonstrated by Onodi,[3] that the sphenoidal sinus, or the posterior ethmoid cells of *one side*, may be in direct relationship with the optic nerve canal, or optic chiasma, of *both sides*, being separated by a shell of bone as thin as paper; and thus one may explain cases where one-sided ethmoidal or sphenoidal empyema has caused blindness or other ocular disturbances on the *opposite* or healthy side of the nose.

Skin affections are practically limited to the nose, but are worthy of consideration. So-called recurrent erysipelas is sometimes due to ethmoid cell suppuration, and in other cases less definite attacks of recurrent œdematous inflammation arise, and the tissues of the upper lip and nose may become chronically infiltrated and thickened. The nasal symptoms may be slight

[1] *Med. Rec.*, 1906, lxx. 689.

[2] *J. Am. M. Ass.*, 1899, xxxiii. 1197.

[3] *Trans. of the Congress of Ophthalmology*, Heidelberg, 1906.

or obvious enough on examination, but in a few cases I have observed the nasal secretion to be muco-purulent.

In one case a young girl completely recovered after operations on the ethmoid region and the maxillary antra (Fig. 11); and in another (Fig. 12), a boy, treatment by anti-streptococcal serum injections was suggested but refused.

Fig. 11.

*Nasal hypertrophy due to nasal accessory sinus suppuration.*

Fig. 12.

*Recurrent inflammatory œdema of lips due to purulent nasal disease.*

Another patient still under my care suffered for many years from his nose, which persisted in getting red and swollen, and despite all abstemious habits and quiet life, it was a constant and perpetual source of annoyance and humiliation to him The lower half of the nose was doughy, with uneven surface, with fine venules coursing over it.

### "NASAL MUCOUS POLYPUS"

What is the connection between the so-called "nasal mucous polypus" and nasal suppuration? Firstly, the common mucous polypus of the nose is not a myxoma, but is a localised œdematous fibroma, being composed of fine meshes of areolar tissue, filled with fluid containing serum albumin with a trace of mucin, and it is covered with the ciliated epithelium of the mucous membrane while small, although this normal covering is often lost and replaced by stratified epithelium as the polypus becomes larger. Various theories have been propounded from time to time to explain the origin of polypi, but none appeared to me to accord with the clinical facts. Writing in 1891,[1] I therefore advanced the view that probably obstruction arises in the lymphatic vessels, owing to the invasion of micro-organisms.

On Plate I., Figs. 3 and 4, I show microscopical sections, prepared by Prof. Walker Hall for me, from a patient operated on for nasal polypi and antral and ethmoidal suppuration. Gram-negative and Gram-positive small granules are seen forming aggregations in masses around a nucleus, and some strings of isolated granules are also observable. It seems probable that these masses are mast cells which very rarely occur in polypi. I think the origin of some polypi is as follows. The pathogenetic cocci invade the epithelium, enter the lymphatic spaces, and are carried to the small lymphatic vessels in which, with or without consequent endo-lymphangitis, they cause blocking of the lymphatics. The blood vascular supply remains unaltered, and the very active secreting functions of the affected mucosa persist, while the fluid poured into the lymphatic spaces fails to be removed, and accumulates in that implicated area. If this occurs close to the surface, a corresponding elevation of the epithelium is seen to protrude; if deeper in the mucosa, a pale area of œdematous connective tissue arises. In either case, as the accumulation increases, the area corresponding to the blocked lymphatic steadily increases till a minute polyp begins to protrude, and becomes a small mucous polypus. If the supply of infecting material ceases, the arrested lymphatic circulation may re-establish itself and the polyp may disappear; but if the lymphatic vessel is permanently blocked, the polyp continues to grow till it may attain enormous proportions. Inasmuch as the area involved for any particular polyp, as far as the surface of the mucous membrane is concerned, is the restricted territory of the involved lymphatics, the polyp, however large, has a relatively narrow pedicle of origin. Such polypi form in the mucous membrane

[1] *Diseases of the Nose, Pharynx, and Larynx*, 4th Ed., p. 325.

of the outer wall of the nose, above the inferior turbinal, and in all the accessory sinuses.

As nasal polypi arise in this manner, they are always more or less pedunculated when of any considerable size, and hence are usually best removed by picking them off at their attachment instead of cutting through the body of the polyps by a snare (Fig. 13).

Fig. 13.

*The Author's forceps shown in situ removing polypi at their narrow base.*

The presence of multiple polypi in the nose is strong evidence of the existence of suppuration in one or more of the accessory sinuses, and it is not difficult to understand that infective pathological processes such as I have described may afford sufficient peripheral irritation to set up not paroxysmal sneezing only, but the more troublesome neurosis, paroxysmal asthma.

I have only sketched in the briefest manner possible what I consider is the usual infective origin of nasal polypus, reserving for a future occasion any reference to the many other points that demand consideration, such as the differences between single and multiple polypi, the relationship of bone changes to those observed in the mucosa, why polypus is usually associated with ethmoid cell disease, the nature of aural polypi, and so forth.

Plate I.

Fig. 1.

*Photo-micrograph of section showing a projecting localised œdematous infiltration with a distinct pedicle, i.e. a commencing polyp (1 inch obj.).*

Fig. 2.

*Section showing (in centre) area of marked cell infiltration surrounded by œdematous area (1 inch obj.).*

PLATE I.

Fig 3.

*Section of œdematous polyp showing small masses of Gram-negative granules. Thionin stain. (Obj. $\frac{1}{10}$.)*

Fig. 4.

*Section of œdematous polyp, showing small masses of Gram-negative and Gram-positive granules. Gram stain. (Obj. $\frac{1}{10}$.)*

### TREATMENT.

Before referring in any detail to the question of treatment, I desire first to emphasise two points of cardinal importance: firstly, that our standpoint should not be too local; and, secondly, that in acute or chronic suppuration of the nose or ear, general surgical principles of free exit to the pus, and free drainage, must be our guiding principle, an axiom that scarcely needs elaboration.

Consideration of local conditions of suppuration in the nose does not alone suffice for successful treatment of tuberculous or syphilitic disease or chronic diphtheria of the nasal passages, but the importance of general treatment is less recognised when the accessory sinuses are implicated. But here, too, a tuberculous or syphilitic infection, for instance, may baffle every effort to effect a cure by local methods alone. One case in particular I call to mind, that of a medical man who came to me from India on account of a persistent frontal sinus empyema. The symptoms seemed to date from the time when he had malarial fever, and remembering that chronic synovitis of a joint may be caused by malaria, I put him on a course of quinine, with unexpected and remarkable success, for apparently the undoubted sinus trouble subsided and disappeared.

**In acute accessory sinus inflammation** there is no need for special treatment, unless as a consequence of the swelling of the inflamed mucous membrane the openings into the nose become blocked, and the profuse secretion retained. The frontal headache and noseache of a simple acute catarrh, and of influenza, measles, &c., is due to acute inflammation of the lining mucous membrane of the accessory sinuses, but when these become intensely acute and localised, it is due to retention of the secretion. In such conditions temporary relief is obtained by hot fomentation externally, while a fine spray of adrenalin and cocaine, or of menthol and cocaine, directed well up the nasal passage may cause the mucosa of the ostia to shrink sufficiently to allow the muco-pus to escape. Ten drops of a saturated solution of menthol in rectified spirit added to a pint of hot water, and the steam inhaled through the nose, may have the same beneficial effect.

Again, the use of suction by Sondermann's suction mask applied over the nose may induce the apertures to open.

Failing relief in this manner, or even when the affected sinus secretions do periodically escape, the danger of an acute sinusitis becoming chronic should be borne in mind, and if such an accessible cavity as the maxillary antrum be the seat of trouble, it is much better to make a small opening through the alveolus or canine fossa, so as to allow the cavity to be irrigated daily till it becomes healthy.

### TREATMENT BY OPSONIC VACCINES.

**In chronic open suppuration** from the accessory sinuses, the fact that it has become chronic indicates a deeper lying and more persistent infective process than when it is an acute stage, but even here, with free exit to the discharge, the infected mucosa may regain its normal condition. The same applies to purulent otitis media, the essentially different condition being that there is practically no normal exit, and nature or art has to make one through the tympanic membrane, and we often have to aid the exit of the secretions by intra-tympanic irrigations or by removing the outer attic wall.

But we have at our disposal a method which is still on its trial, but from which it is reasonable to hope we may derive great assistance in obviating the necessity of radical operations.

I have resorted to vaccine treatment in cases of frontal sinus and ethmoidal cell suppuration, and in chronic purulent otitis media.

My first case I was attending in conjunction with Dr. Bruce Kelly of Burnham, a patient with one-sided antral and fronto-ethmoidal suppuration. A radical antral operation with partial removal of the middle turbinated body was performed with very satisfactory results as far as the antrum was concerned. Frequent irrigation of the frontal sinus had considerably improved matters there, but the purulent discharge continued to be copious and gave no indications of clearing up. In April we sent a specimen of the pus from the frontal sinus to Dr. Munro of Bath, and the results of his cultures and of the injections of vaccines were as follows :—

April 7th, 1907.—Staphylococcus in pure culture in pus drawn from frontal sinus. Tubercle bacillus not found. O.I. to staphylococcus 0.81. O.I. to tubercle bacillus 1.06.

April 17th.—First injection of vaccine. (100 millions staphylococcus.)

April 24th.—O.I. 1.15.

April 27th.—100 millions staphylococcus injected.

May 3rd.—O.I. 1.03.

May 4th.—Injection of 100 millions.

May 11th.—Injection of 100 millions.

May 19th.—O.I. 0.8. Large increase in amount of discharge and pain. Patient weak and ill. Acute exacerbation.

May 19th.—Injection of 100 millions.

May 29th.—Injection of 200 millions.

June 9th.—O.I. 1.5. Injection of 500 millions staphylococcus. Discharge much less. Patient feeling much stronger, very little pain.

June 19th.—Injection of 500 millions. Continues to have very little discharge or pain, and feels very well.

July 7th.—O.I. 0.95. Injection of 100 millions. Having a good deal of pus down the throat. Slight exacerbation.

July 17th.—500 millions injection. Much less discharge.

July 28th.—Injection of 500 millions.

Aug. 9th.—Injection of 500 millions O.I. 1.2. Patient feeling well, very little discharge.

Aug. 29th.—Injection of 500 millions.

Sept. 15th.—Injection of 500 millions. Not feeling so well, getting thinner, culminated in an acute exacerbation on September 23rd with great pain and much discharge. Sinus washed out on September 30th. No more pain. Discharge gradually got less.

Oct. 7th.—Injection of 200 millions only, it having been found that 500 millions produced invariably a negative phase with increased pain and discharge lasting about a week.

Oct. 21st.—Injection of 200 millions O.I. 0.8. Patient then went to Channel Islands, and there has not been an injection since.

In January, 1908, Dr. Kelly reported that the patient says the headaches are getting much less, that they are not so violent when they come, that she does not have the "cotton-woolly" feeling now, and that she feels better, locally and generally, than she has done for a long time—perhaps years past.

One case in which resort to this method was apparently of great service was that of Mrs. R., aged 36, who had chronic purulent otitis, with perforation of Shrapnel's membrane, as well as a large opening in front of the handle of the malleus. Removal of the attic wall and frequent irrigations had improved, but failed to cure. There was no direct evidence of any necrosis of the ossicles or tympanic wall, but it was fairly certain that the mastoid antrum was the source of suppuration.

Mrs. R.—Colonies of staphylococcus pyogenes aureus and albus were isolated from the ear discharge. From each of these growths a separate vaccine was made on July 6th and standardised. These were mixed and injections given, commencing with 250 millions.

July 25th.—O.I. No. 1 organism 0.8. No. 2 organism 1.47.

After four injections the opsonic index to each organism was taken on October 8th, 1907.

No. 1.—O.I. to own staphylococcus aureus 0.58.

No. 2.—O.I. to own staphylococcus albus 0.60.

They were almost identical, but still low. She was then completely free from all aural suppuration, and has remained so.

In another case where, following a Delasaeux radical operation on the frontal sinus, some purulent discharge continued for a lengthy period, the culture from the pus proved to be unmixed streptococcus brevis. A first injection of the vaccine, of 20 millions, was given. This was followed by diminution, and finally by disappearance, of all discharge without any further treatment. Unfortunately, no indices were taken before and after the vaccine injection.

In another case of frontal sinus suppuration I was not able to trace any improvement from the vaccines. How far the improvement in these cases is attributable to the vaccines it is as yet unsafe to say, but, at any rate, the results were sufficient to encourage resort to the method in certain cases. I think that probably smaller injections more frequently repeated would give better results and avoid long negative phases.

### MAXILLARY ANTRUM SUPPURATION.

When the diagnosis of antral suppuration has been made, we have to decide on the question of operation : shall it be a simple trephining of the alveolus through the canine fossa, or entry through the inferior meatus by the nasal route, so as to allow a daily washing out of the antrum ; or must a radical operation be performed ?

Undoubtedly many cases of not too long standing may be cured by the simple operation, followed by irrigation. If teeth corresponding to the floor of the implicated antrum have been lost, or one is so decayed as to warrant its extraction, the alveolar route has certain advantages, the chief of which is that it is easy to perform I know of no alveolar method that can take precedence of Mr. Ackland's.

By his instrument, a hole is bored into the antrum, and with the introducer, a very ingeniously-devised tube with a retaining screw-thread which retains it in position, is readily screwed home. It has a split pin stopper, which the patient can remove to attach the well-fitting syringe nozzle, and when the irrigation is completed the patient can himself replace the stopper so as to keep out any food or saliva from the mouth.

Usually I prefer opening with a quarter-inch trephine through the canine fossa, preceded by incision in the mucous membrane and reflection of the periosteum over the area to be trephined. It can be done under gas anæsthesia. I have had self-retaining antral rubber plugs made, and one of these is slipped into the hole. Either then, or a day or two later, the antral cavity is inspected by means of a good light thrown in through an antral inspecting tube, a method elaborated by Kelly, of Glasgow. Every part of the inner wall and roof can be seen, and, in fact, portions of the other walls. The amount of thickening of the lining membrane, the existence of polypoid degeneration, and especially the condition of the antral mucosa about the unciform process and middle turbinal region, can be accurately observed, and one can often determine how far a good result from simple irrigation is possible, or whether nothing short of a partial curettement of the antrum, or even a partial removal of the inner wall, is a *sine qua non*. Otherwise the patient may have to go on washing out the antrum daily for some months in a futile hope that a radical operation can be avoided.

To make an aperture in the inferior meatus such as will enable the patient to irrigate the antrum is certainly much more difficult, and when it has been accomplished one cannot inspect the inner wall of the antrum at all, and the other less important parts of the cavity can only be very imperfectly inspected. Of late this intra-nasal route has been advocated, or may I say its advocacy has been revived, for John Hunter was the first advocate, although he proposed entry through the middle meatal wall.

If this route be selected, we have at our disposal various trephines, short strong angular knives, or cutting forceps.

I formerly used a cutting forceps of my own pattern, which was efficient as a means of entering the antrum and cutting a hole that sufficed for irrigation. My colleague, Mr T. Carwardine, has introduced a very ingenious set of cutting forceps, by means of which the opening made in the outer nasal wall can be gradually enlarged by clipping away the bone wall upwards, backwards, downwards and forwards, so as to make an opening well down to the floor of the nasal passage.

I know of no *intra-nasal* method which can compare with this operation of Carwardine's, for the amount to be removed is completely under the control of the operator, while the irregularity of the margins of the opening is certainly not any greater than when a hammer and chisel are used in the usual way through the canine fossa in the Ogston-Luc operation.

My later method, however, appears to me to possess advantages which outweigh all that can be urged in favour of the nasal route. I simply trephine a sixpenny-sized opening in the canine fossa, exactly in the same way as the small trephine opening is made for inspection and irrigation. The trephine, made for me by Mayer and Meltzer, has a special gimlet-like pin, so that the

Fig. 14.
*The author's operation on the maxillary antrum, slightly reduced.*

Fig 15.
*To show the relative size of the permanent opening after healing.*

circle of bone is retained within the trephine. Every part of the cavity is readily and accurately inspected, and one can at once see how much of the lining mucosa must be curetted and how much of the inner wall must be removed. Often one can thus avoid doing anything like a complete radical operation, while at the same time ensuring the complete removal of all polypi and diseased areas of bone, which is essential to successful results. The same trephine is then passed through the opening in the canine fossa till it is pressed against the portion in the anterior part of the lower meatus which is to be removed. The tip of the forefinger of the free hand is passed into the nose, so that the septum is protected as the trephine cuts through into the nasal passage. In this way *a perfectly round opening with smooth edges* is made to connect the anterior and lowermost part of the antral cavity with the nose flush with its floor, no matter how thick the bony wall is at that point. The disc of bone and the corresponding piece of the inferior turbinal comes away in the trephine. It is a relatively easy operation, and ensures the least possible removal of healthy tissue ; in fact, after the parts have healed, it is often very difficult by simple inspection of the nasal passage to see that anything has been done.

Fig. 16.

*The author's post-nasal plug and tongue-hook for operations in the nose.*

The ½-inch opening gets smaller when the edges epithelialise over, but it never closes ; whereas a smaller trephine opening contracts, and sometimes closes altogether.

The opening into the mouth quickly closes, and after the first dressing all irrigation is carried out through the nose. One of the worst cases I have had to do lately—a double radical antral operation with complete curettement of polypoid antral mucosa, and very extensive removal of the ethmoid cells and polypi and the unciform region—was out for a walk on the tenth day, and was trying his hand at golf on the fourteenth.

In cases requiring radical operation there is not infrequently greater pain and swelling and general febrile disturbance, with quite a minor procedure, than with the much more extensive radical operation. This particular patient came to me for nasal obstruction, and though I carefully examined him for accessory sinus disease, I could not satisfy myself of its existence ; for not only was there none to be seen at the time of examination, but he assured me he never had pus or discharge from the nose anteriorly or posteriorly. The antra transilluminated well, although there was no pupil reflex. When I operated on the deflected septum by submucous resection, I also clipped away a rather full, boggy anterior end of the left middle turbinal. The nasal obstruction was removed, but the following day the left upper and lower eyelid was œdematous. On examining the nasal passages, a quantity of pus could be seen in the middle meatus on either side, and in the upper ethmoidal region. The orbital cellulitis culminated in an orbital abscess, which was opened by Dr. Ogilvy. The patient was three weeks in the home, and he remembered afterwards that he used to be frequently swallowing or hawking up purulent and bad-tasting matter. The fact is that in the course of years he had grown so used to it that he did not notice it, and on account of the nasal obstruction all the pus had gone down the naso-pharynx. Yet when, very shortly afterwards, he returned to the home and had the extensive operation he was up and out in ten days.

When I reflect how on several occasions I have nearly missed detecting an accessory sinus empyema when examining a nose, I can only urge that oftentimes the patient's perceptions seem so blunted that the history given seems hardly compatible with the actual state of affairs.

A very striking example of similarly misleading symptoms and signs in aural troubles occurred to me some time ago, when I was asked to see, in consultation, a lady in Paignton, aged 56. Amongst other troubles, she had had purulent discharge from the right ear, off and on, since girlhood, and a few days previously had complained of pain in the ear, with slight rise of temperature.

The day before I saw her she had been suffering great pain, especially in the front of the meatus, but also all over the right temporal region. This had been relieved by morphine, and had subsided, but the pain in the meatal region remained fairly acute. She was quite clear and even fairly bright when I saw her, and had no headache. The question arose as to whether an abscess in the anterior meatal wall accounted for the pain and febrile disturbance. There was no headache, the optic discs were normal, except that the retinal veins were doubtfully full. There was no redness, œdema, or tenderness over the mastoid, and very firm pressure over the antrum only elicited the expression, " Oh, how nice ; it gives a sense of relief." I confess I felt that the symptoms rather pointed to an intra-meatal abscess, as the meatal wall was swollen and red, especially posteriorly, but an incision showed that no collection of pus was there. She had been prepared for opening the mastoid, and an ordinary radical mastoid operation was performed, a large antrum full of stinking pus being cleared out, together with the mastoid cells. There was nothing abnormal about the emissary vein, in the absence of all grosser signs one of the surest indications of septic lateral sinus. But having completed the operation, careful search was made for any patch of softened bone, and at the roof of the antrum the searcher passed into the cranial cavity, and a large quantity of fœtid pus streamed out. The opening was enlarged so as to allow very free drainage of the subdural abscess. Everything went well, and she improved in every way till the third day, when I heard by telephone that the temperature and headaches had returned, and the patient showed other indications of intracranial mischief. I suggested that fine, blunt-pointed forceps should be introduced in the direction of the temporo-sphenoidal lobe, and when it had entered $1\frac{1}{2}$ inches a quantity of pus escaped. Although for a short time the evacuation of this temporo-sphenoidal abscess was followed by improvement, the patient never rallied. There can be no manner of doubt that, despite the remarkable absence of symptoms, this patient's mastoid suppuration had set up a latent subdural and temporo-sphenoidal abscess, and that she was getting about in her usual way at a time when there was no suspicion of any such grave condition existing.

### FRONTAL SINUS SUPPURATION.

Irrigation of a suppurating frontal sinus may sometimes succeed in curing the condition, and is usually worthy of trial, not because it is likely to be successful, but because the radical operation is dangerous, and one can never tell beforehand how much deformity may result, or how many complicating factors may be revealed only after the operation is begun. There is very good reason why irrigation is rarely successful, in that the frontal sinus, often as it is the seat of inflammatory disease and suppuration, generally drains and cures spontaneously, owing to the favourable position of the fronto-nasal channel for drainage, *unless anatomical conditions make this impossible.* Thus, if the anatomical conditions permit of irrigation, the occurrence of chronic sinusitis is relatively rare, and where, with a fairly patent fronto-nasal duct, natural drainage has failed to prevent the sinusitis becoming chronic, it is usually due to pathological conditions which will render irrigation futile. Nevertheless, irrigation may succeed, and I have had completely successful results in more than one case.

**Radical Operation.**—The design of the modern radical operation is to remove the whole of the diseased tissues of the frontal sinus, and the fronto-ethmoid cells, to secure free drainage into the nasal passages, and to obliterate the frontal sinus with as little disfigurement as possible. The radical operation has been widely practised, and the results justify its adoption in all cases calling for interference when conservative methods have failed to give sufficient relief. The earlier radical operation left such great deformity, that in this country at any rate it was never received with favour, except as a last resort. To Killian, of Freiburg, belongs the credit of initiating the cardinal point in the modern operation, viz. the *preservation of the supra-orbital bony margin*, by which the facial defect is lessened to a very great extent, so as to be even trivial in favourable cases.

The essential features in Killian's operation are shown in the plate, No. II. He saves the supra-orbital margin of bone, and the bridge extending to the root of the nasal bone, but removes the whole of the rest of the anterior wall of the sinus *and the floor*, making a free opening into the nasal cavity by removing the portion of the bony wall below the bridge and in front of the lachrymal groove, and clearing away diseased ethmoid cells even when necessary right back to the sphenoidal sinus.

At the edge of the nasal bone perforate the nasal membrane with a pointed scalpel. By means of a probe-pointed scalpel, continue the incision upward and backwards ½ c.m. below the lamina cribosa, then downwards. This flap of nasal membrane is turned outwards, and is used to cover those parts of the wounds facing the nasal cavity. A wide communication between nasal cavity and frontal sinus is permanently secured.

Killian then allows the soft tissues of the supra-orbital flap to sink back against the posterior wall of the sinus, and the orbital fat bulges up to complete the closure of the gap left by removal of the sinus. He leaves the inner third of the wound unstitched, and packs the cavity through it till it is closed by granulation.

In Great Britain Tilley adopts a modified Killian's procedure, whereby he has been enabled to greatly shorten the subsequent stages of healing. He does not remove the floor of the sinus forming the orbital roof, except so far as to make a free passage into the nose and remove all implicated ethmoidal cells; but having, like Killian, removed all the diseased mucous membrane of the sinus, cleared away all septa that could interfere with drainage, and followed up and cleared away any small pocket that contains pus, he sews up the external wound at once.

Delsaux has devised a modification of Moure's operation for the removal of malignant growths of the ethmoid, which he resorts to for the radical treatment of multiple sinus suppuration involving the frontal sinus, the ethmoid cells, maxillary antrum, &c. An incision extending down to the bone is made, extending from the centre of the brow along the internal angle of the orbit, descending on the lateral face of the nose till it reaches and terminates at the corresponding naso-labial depression. The periosteum is divided and detached upwards and downwards. Then the soft tissues over the frontal bone are detached, and the frontal sinus is opened with a gouge through the floor close to the nasal spine. The whole or part of the inferior and anterior walls of the frontal sinus are removed, according to the necessities of the case. The nasal bone and the ascending process of the superior maxillary bone are now removed, without at this stage opening through the nasal mucosa, so that the blood will not encumber the nasal passages. Of the anterior and middle ethmoidal cells sufficient are removed to ensure good drainage from the frontal

### Plate II.

**Fig. 1.**

*Killian's radical fronto-ethmoidal sinus operation, showing the bridge.*

**Fig. 2.**
*Author's case. Operation on the ethmoidal cells, which were cleared away right back to the sphenoidal sinus, which was opened. Operation on right side, as in the Killian operation. The frontal sinus did not exist.*

**Fig. 3.**
*Delsaux's operation after removal of the frontal sinus wall and of the ethmoidal cells.*

**Fig. 4.**
*Delsaux's operation finished, showing line of incision.*

sinus, and if the ethmoid is diseased that is extirpated too, care being taken to avoid wounding the ethmoidal vessels.

Behind one sees the sphenoidal sinus aperture. If its condition is doubtful this is explored, and if necessary the anterior wall removed. Unless previously removed, one now takes away with cutting forceps the middle turbinated body, and attacks the anterior, superior and inner angle of the antrum of Highmore, and the interior of the antrum is curetted.

Lastly, the nose is pressed back in its place, and one proceeds to suture the divided tissues carefully.

Delsaux has operated thus several times with excellent results, and "very little, if any, deformity." He has operated on two bilateral cases, and then he does not take away the nasal bones, so that, although the operation is less easy, one avoids the deformity that must result if both nasal bones are removed.

**The Author's Method.**—For the radical operation involving the frontal sinus, ethmoidal cells, sphenoidal cells, sphenoidal sinus—as, for instance, in pansinusitis—or in extensive operations on the frontal sinus and fronto-ethmoidal regions for the removal of malignant neoplasms, I have resorted to an osteoplastic operation, which gives very free access to the ethmoidal cells and sphenoidal sinus, as well as to the frontal sinus, and yet avoids the destruction of the nasal bone, and, above all, does not leave a depressed pit below the bridge, for no bridge is made.

The method requires an initial incision extending along the eyebrow towards the root of the nose, thence downwards just outside along the middle line. The skin and soft tissues and the periosteum are raised over the anterior surface of the frontal sinus, the size of which can be determined beforehand by skiagraphy, and the anterior wall of the sinus completely removed to within 3 or 4 mm. of the floor. After removing as much of the floor as seems called for, a second incision, about $\frac{3}{4}$ in. long, along the inner and lower margin of the orbit exposes the lachrymal groove, the duct is then turned outwards, and, with a chisel or burr, entrance is made into the nasal passage. A fine saw, having been passed through the nose so as to come out at this opening, is made to divide the nasal process of the maxillary bone, a second saw-cut being made, extending from the frontal sinus down to the lachrymal groove, dividing the bone from behind forwards, and leaving the soft tissues intact. By making the saw-cut from below through the nasal process of the superior maxillary bone, the facial artery is not divided, and thus the main vascular supply to the flap is not cut off. The first incision is then completed by means of a saw, extending right through into the fronto-nasal duct, and downwards so as to divide the nasal bone *near* the mid-line, but outside the attachment of the septum. The osteoplastic flap is then turned out, giving free access to the fronto-nasal passage and ethmoid cells, which can readily be removed and cleared away if necessary

Fig. 17.

*Diagram of the author's radical operation for frontal-sinus and ethmoidal cell suppuration with an osteo-plastic flap.*

X *Indicates the osteo-plastic flap which is turned back after the nasal saw has divided* Z, *the nasal process of the superior maxillary bone, and* G, *the Gigli saw has divided the bone along the dotted line, in each case from within outwards.* F, *the course of the facial artery, showing how it escapes division and thus secures good vascular supply to the flap.*

right back to and including the sphenoidal sinus. The whole of the pyogenic mucous membrane of the frontal sinus, if it is a case of empyema, is then curetted away and any ridges removed.

### Plate III.

Fig. 1.
*The Author's radical frontal sinus operation, with osteo-plastic flap.*

Fig. 2.
*Case of radical fronto-ethmoidal sinus operation on the left side, two weeks after operation by the Author's osteo-plastic method. From an untouched negative.*

Fig. 3.
*Case of radical fronto-ethmoidal sinus operation on the right side, six weeks after operation by the Author's osteo-plastic method. From an untouched negative.*

The osteoplastic flap is finally replaced and the incisions sutured. In this way it is possible to get a very free access to the dangerous upper ethmoidal region, as well as to the sphenoidal sinus, if necessary.

The mucous membrane of the fronto-nasal duct should be saved, and that on the inner side of the flap be turned back into position with the flap.

After removal of the whole of the mucous membrane of the frontal sinus I have sometimes found it possible to obliterate the cavity by carefully packing it with antiseptic paraffin or wax.

### SPHENOIDAL AND POSTERIOR ETHMOIDAL CELL SUPPURATION.

The posterior group of sinuses, viz. the sphenoidal sinus and the posterior ethmoid cells, offer much difficulty in differential diagnosis, these cavities and their openings into the nose being so close together in the spheno-ethmoidal fissure, far back and high up in the nasal passage, while very often they are all implicated together.

The best plan is to determine first the condition of the sphenoidal sinus, for if either it or the ethmoidal cells contain pus, and the sphenoidal sinus can be excluded, it follows that the ethmoidal cells alone are diseased.

In some few cases it is possible to see the sphenoidal sinus opening through the nasal passage, and then pus may be seen pouring out of the opening, and fresh pus re-appearing as soon as it is wiped away. In a large number a fine cannula can be passed through the opening and the sinus washed out, particularly if the middle turbinal be partially ablated. But, apart from the fact that in most patients such partial turbinectomy is essential before entrance through the natural opening is possible, and thus in the event of a healthy sinus being found an unnecessary operation on the turbinated body is performed, it is not easy to be sure that the fluid escaping from the nose has not become contaminated with muco-pus after its exit from the sphenoidal sinus.

On account of these difficulties and sources of error, I have devised a plan by which the existence of pus in a sphenoidal sinus can be determined with comparative ease and certainty, and I think, providing due care and skill are used, with safety.

**Figs. 18 and 19.**

*The author's sphenoidal sinus cannula and exploring syringe and the sphenoidal sinus cutting forceps.*

Under either local or general anæsthesia, the patient lying on the back, the fairly fine cannula, with a blunt trocar, is passed through the thin anterior wall of the sinus in the following manner.

The blunt trocar and cannula is passed along the floor of the nose till it impinges against the posterior pharyngeal wall; then the distal end is carried forward along the roof of the naso-

Fig. 20.

*The author's sphenoidal sinus exploring cannula in situ.*

Fig. 21.

*The author's sphenoidal sinus cutting forceps in situ.*

pharynx till it slips up the anterior wall. In this way one gets the point about a quarter of an inch above the lower border of the anterior wall of the sphenoidal sinus. Holding the cannula as nearly horizontal as feasible, it is gently but firmly pressed against the thin anterior wall, which it readily enters. The cannula is then removed and the syringe attached. The contents of the sinus are then aspirated, or some boracic acid solution is thrown in, and sucked up into the syringe.

If pus is present, and the sinus has been opened, this is done by introducing blunt-pointed cutting forceps in much the same way, the exact distance of the anterior sphenoidal sinus wall and the depth of the sinus itself having been already measured by the syringe cannula, and as it cuts it is rotated. In this way the sinus could be opened with comparative ease and safety, as it is only the posterior wall that has to bear the pressure of the blunt end of either syringe or forceps, and this wall is always thick and strong.

It is easy to wash out the sinus, if it does contain pus and the anterior wall has been opened, and then if pus still quickly reappears in the region of the spheno-ethmoidal fissure it is certain that it comes from the posterior ethmoidal cells.

An attempt may be made to open up these cells by means of cutting forceps that I devised for the purpose some years ago.

A sufficiently free exit for drainage may in some cases be obtained in this way, but this method failing, the only sure method of reaching these cells is by the external operations I have already described.

## Figures and Tables

**Fig. 1 f1:**
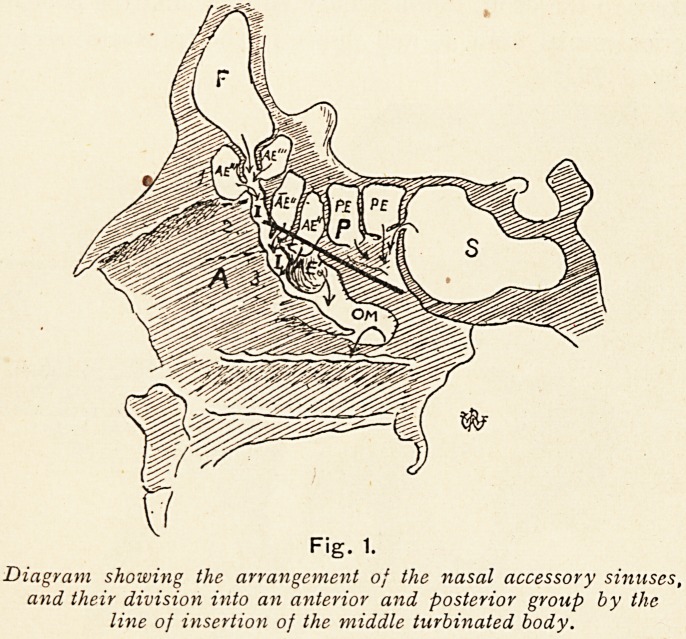


**Fig. 2. f2:**
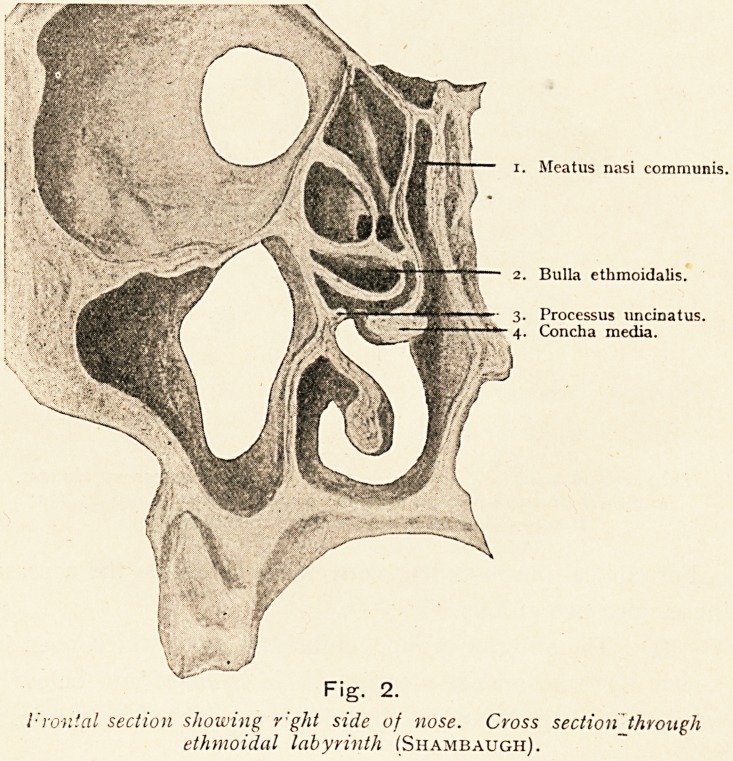


**Fig. 3. f3:**
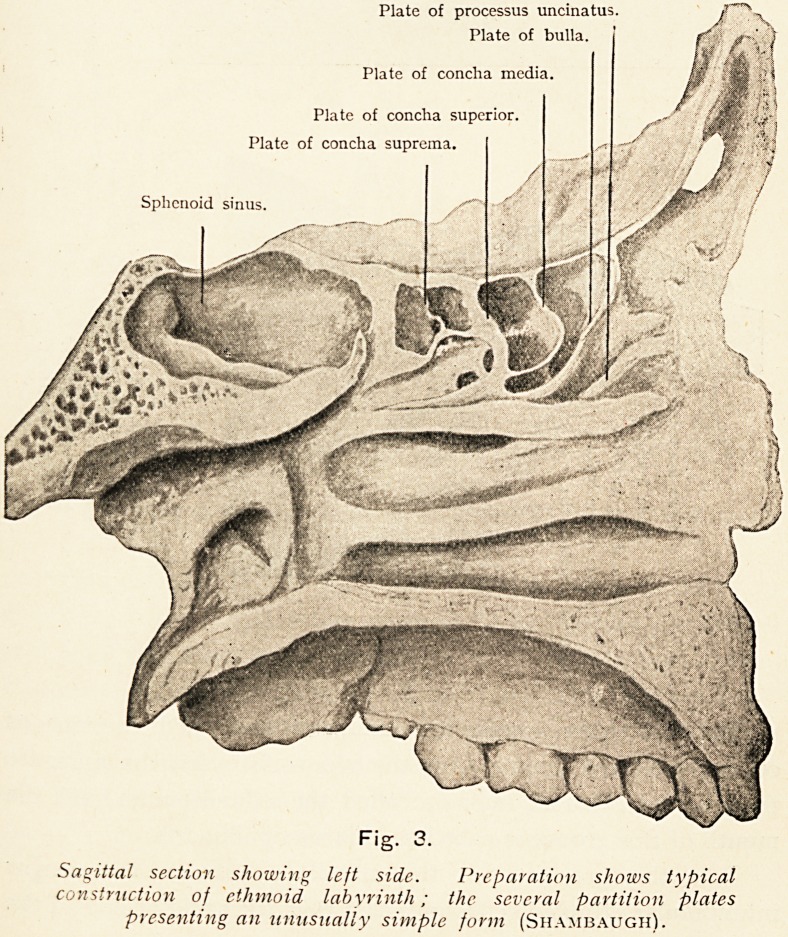


**Fig. 4. f4:**
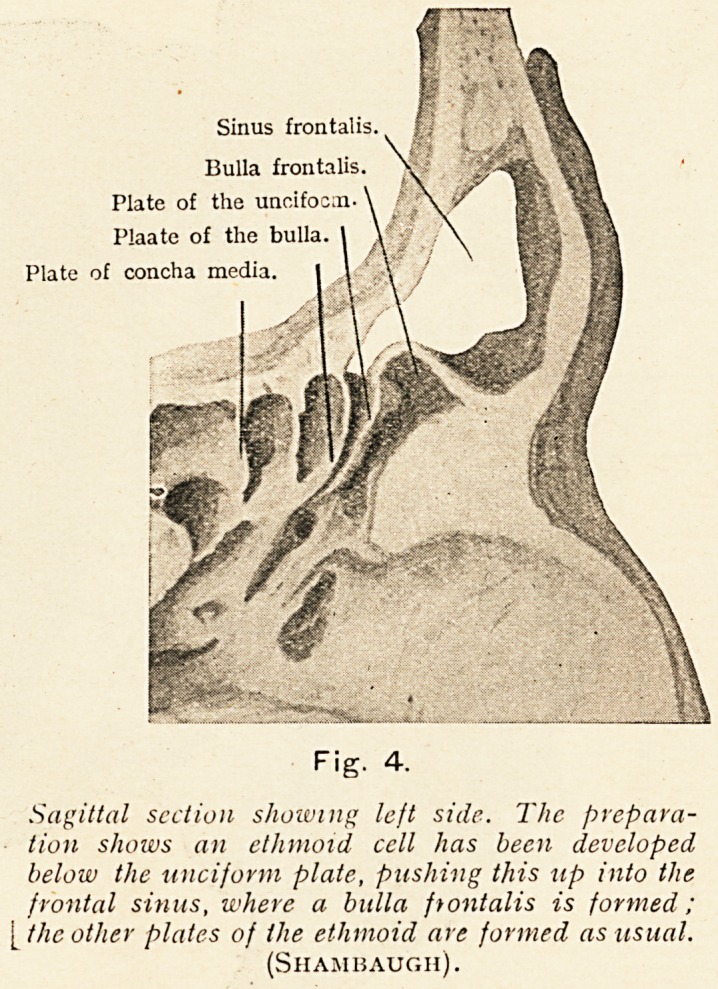


**Fig. 5. f5:**
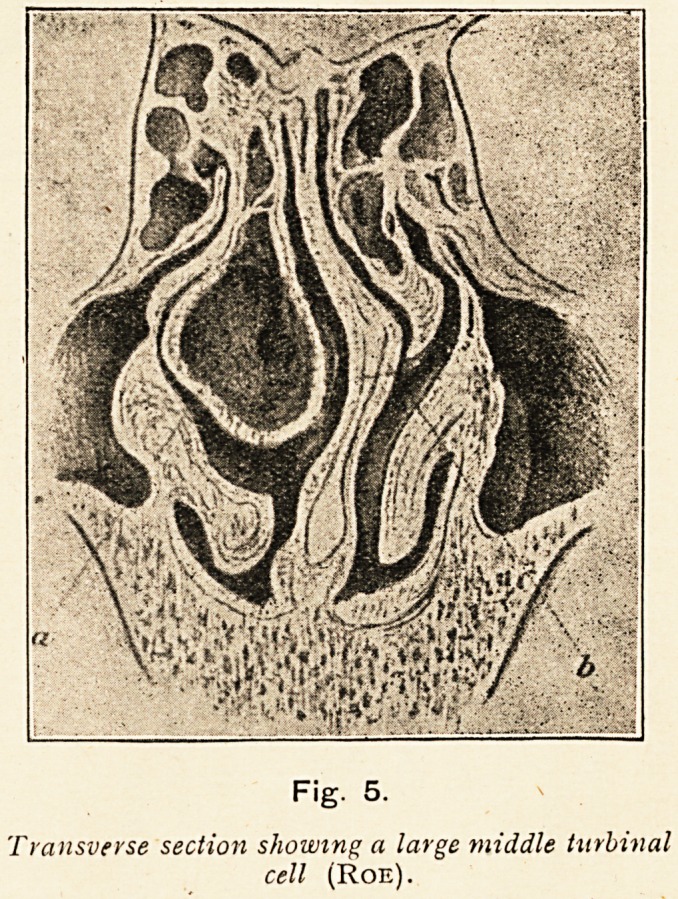


**Fig. 6. f6:**
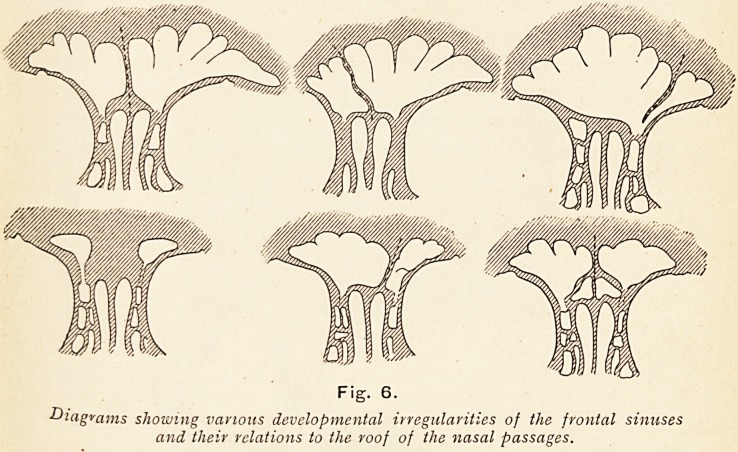


**Fig. 7. f7:**
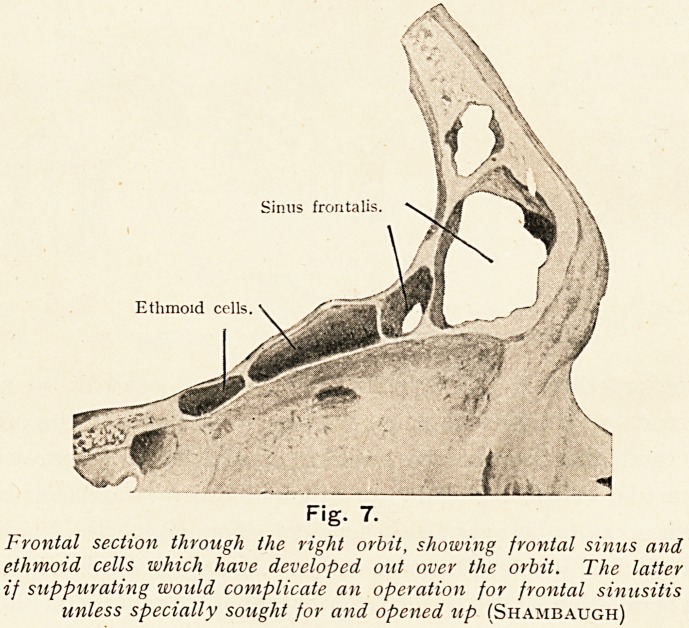


**Fig. 8. f8:**
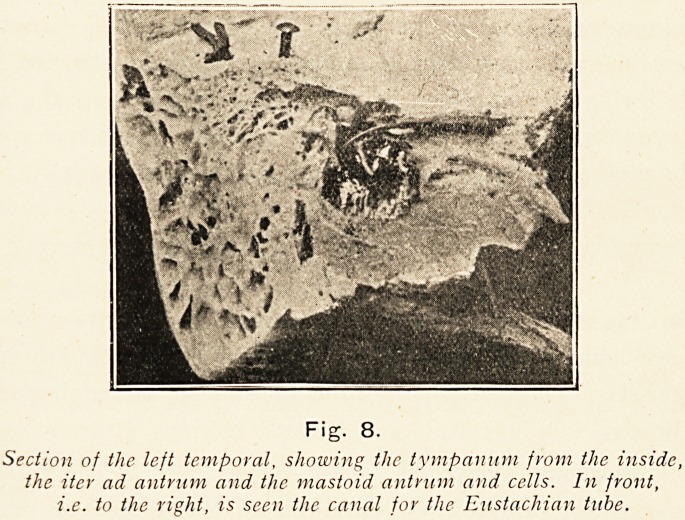


**Fig. 9. f9:**
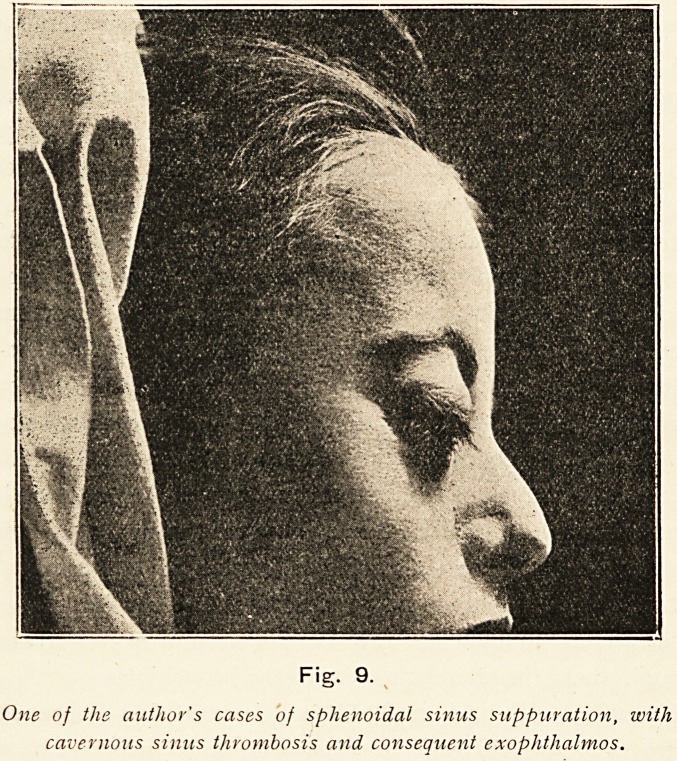


**Fig. 10. f10:**
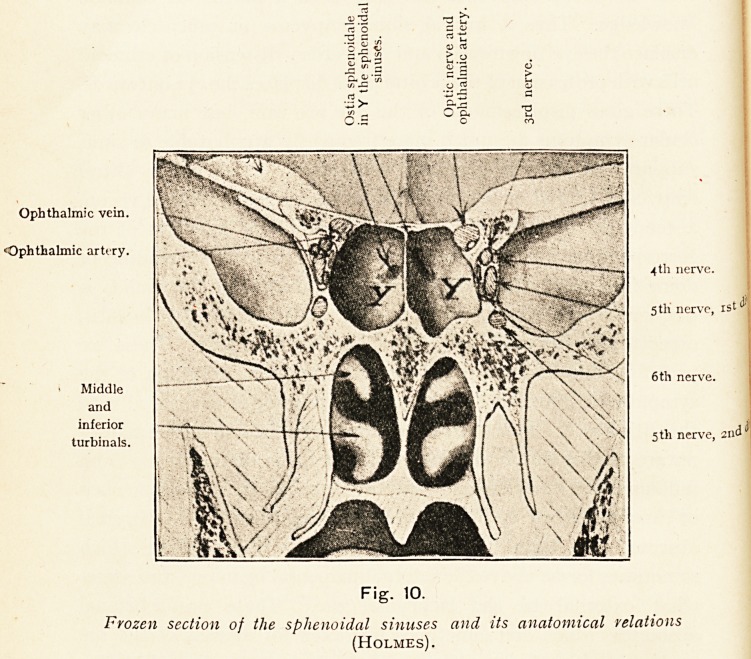


**Fig. 11. f11:**
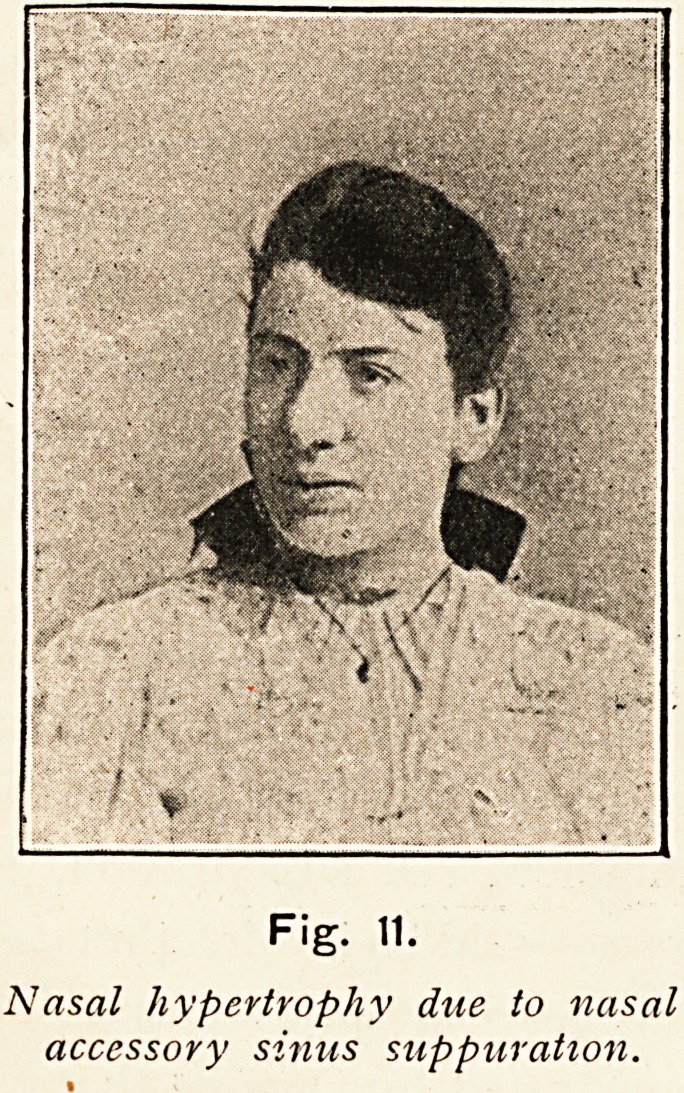


**Fig. 12. f12:**
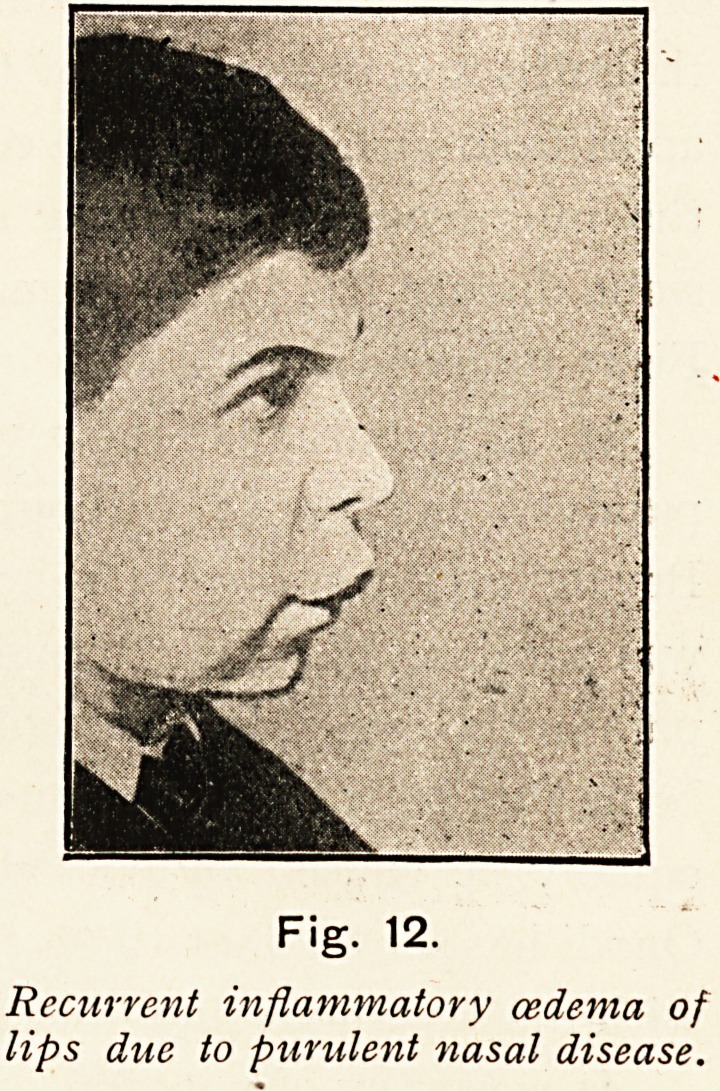


**Fig. 13. f13:**
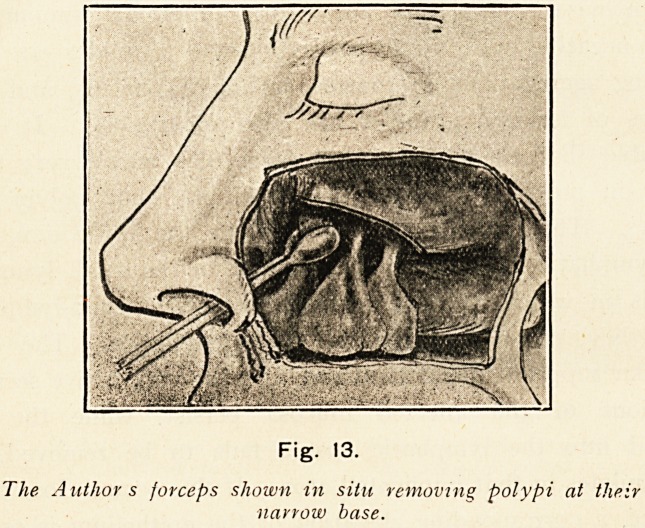


**Fig. 1. f14:**
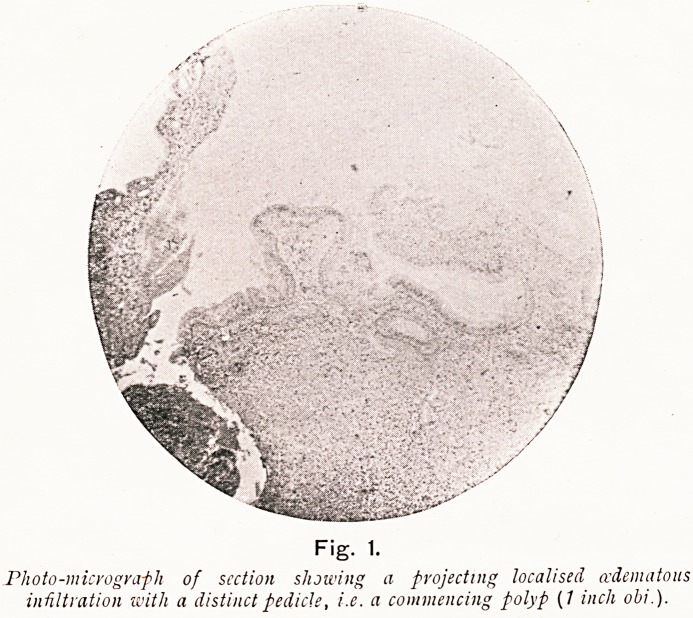


**Fig. 2. f15:**
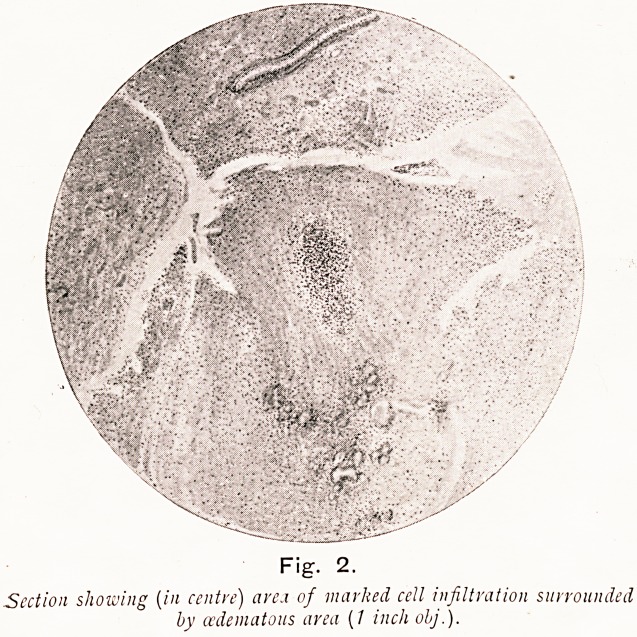


**Fig 3. f16:**
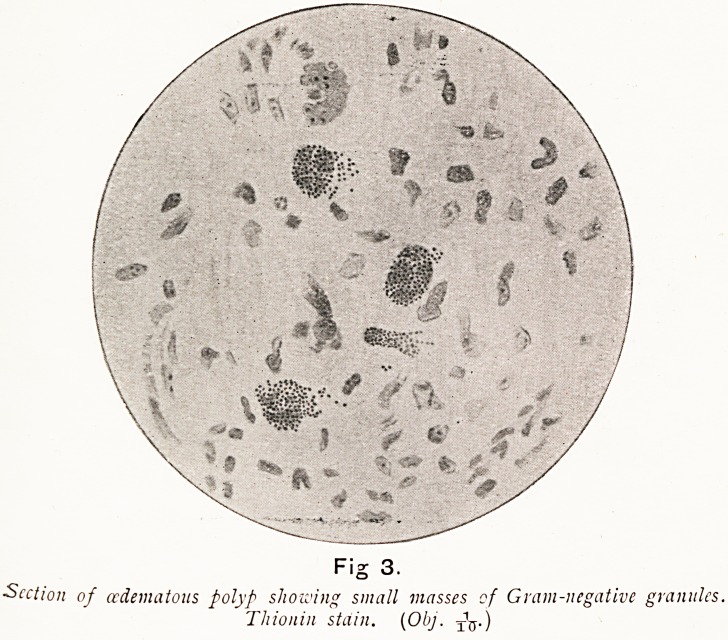


**Fig. 4. f17:**
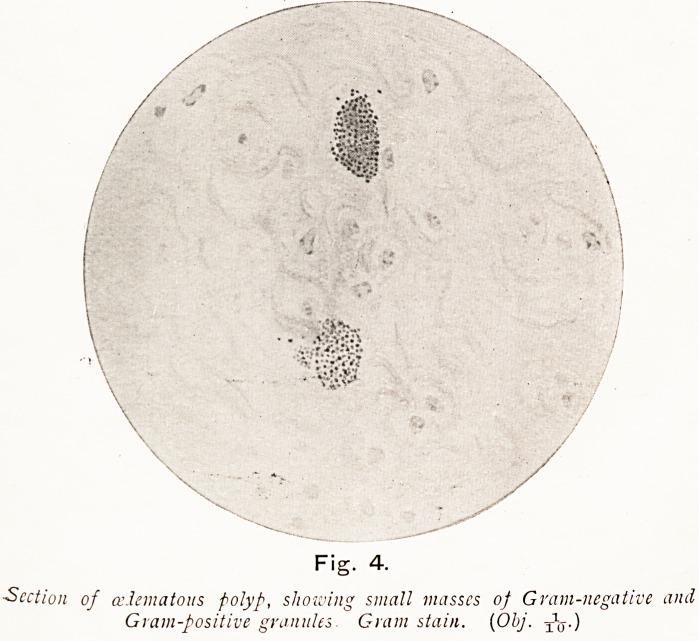


**Fig. 14. Fig 15. f18:**
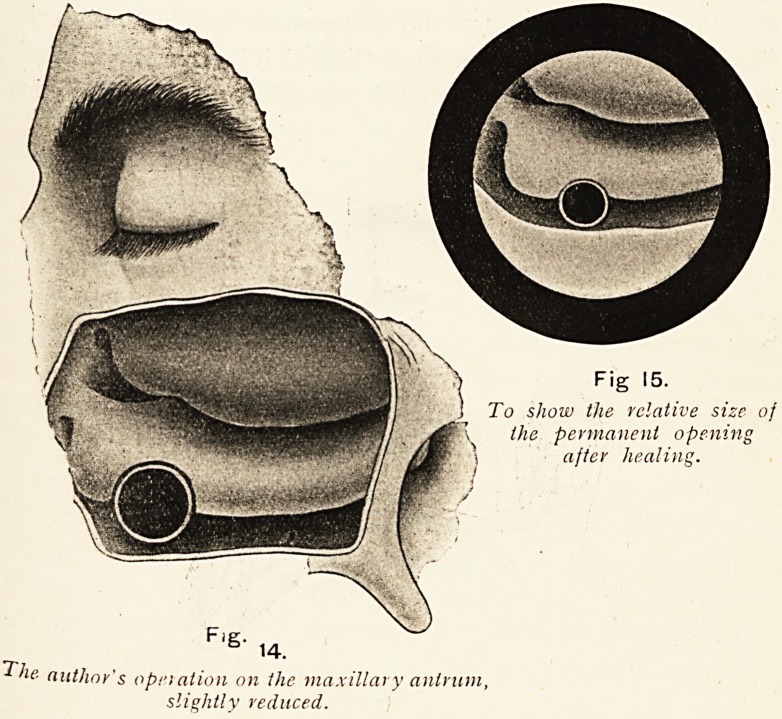


**Fig. 16. f19:**
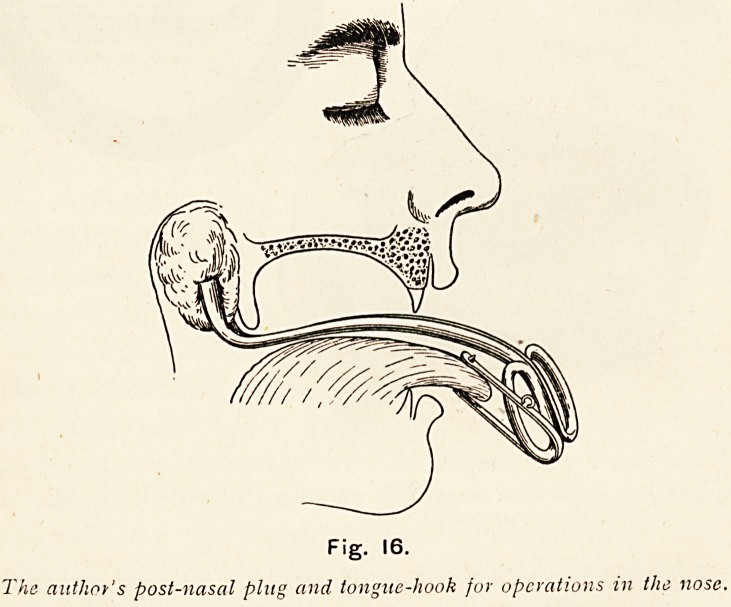


**Fig. 1. f20:**
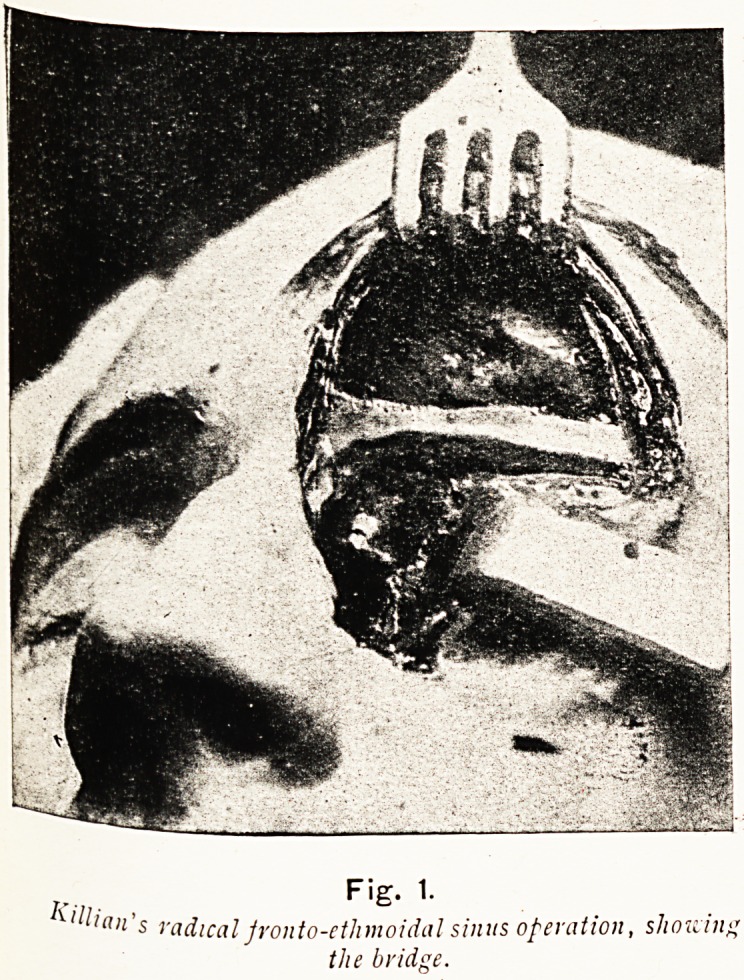


**Fig. 2. f21:**
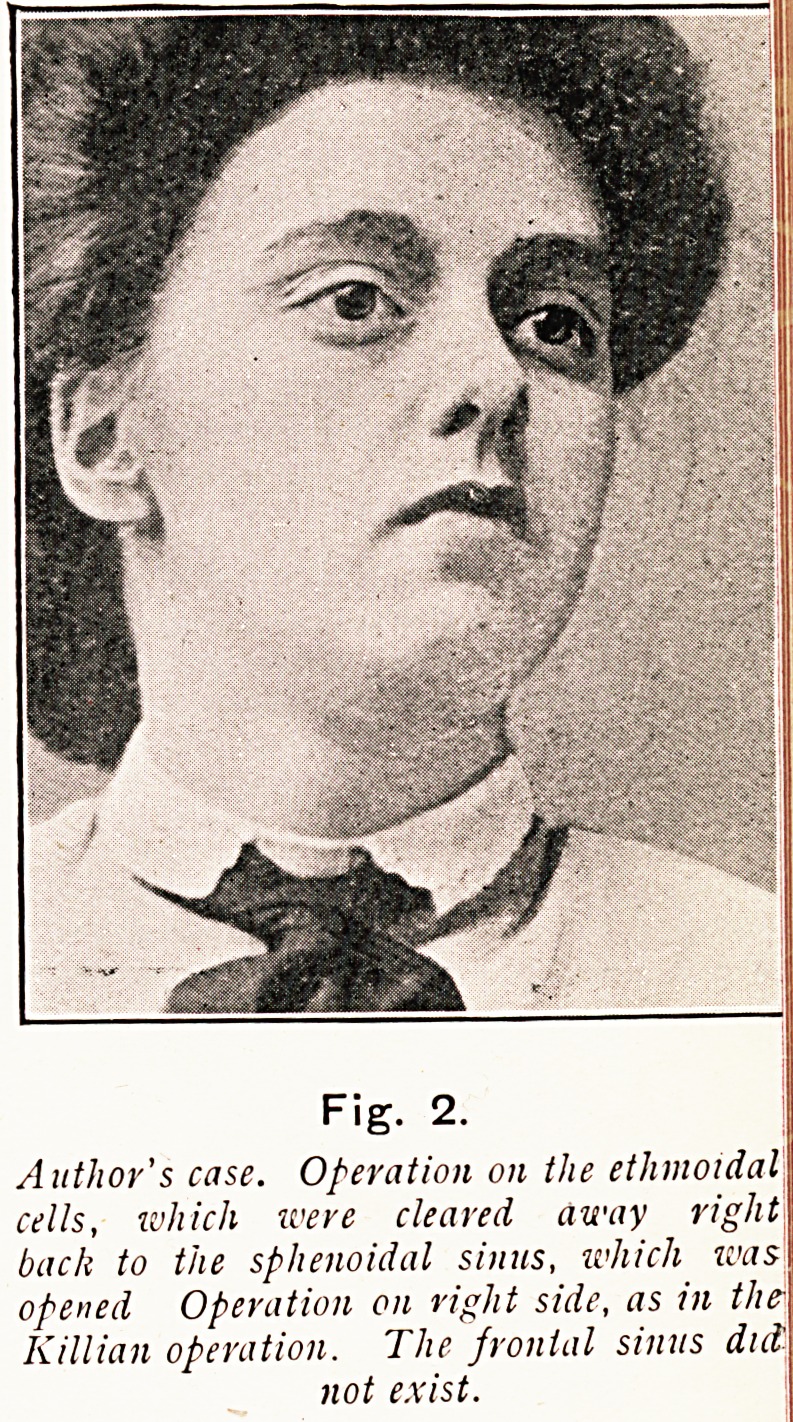


**Fig. 3. f22:**
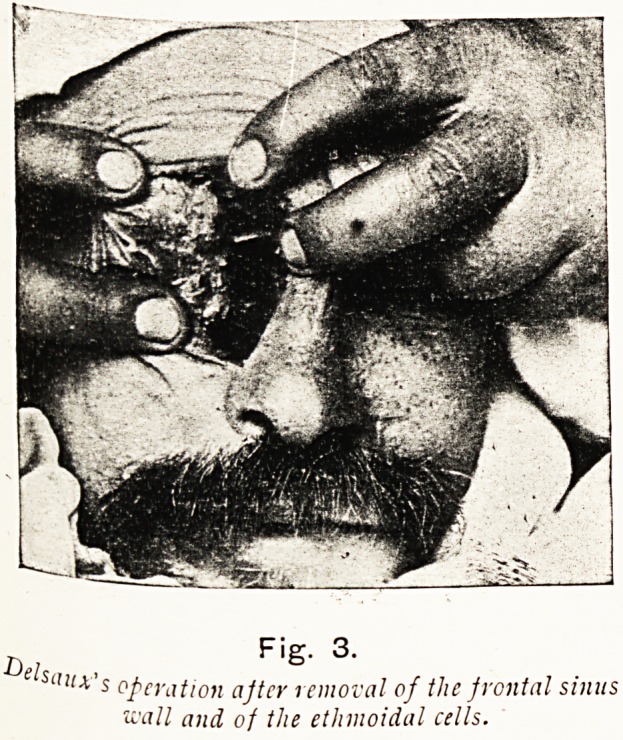


**Fig. 4. f23:**
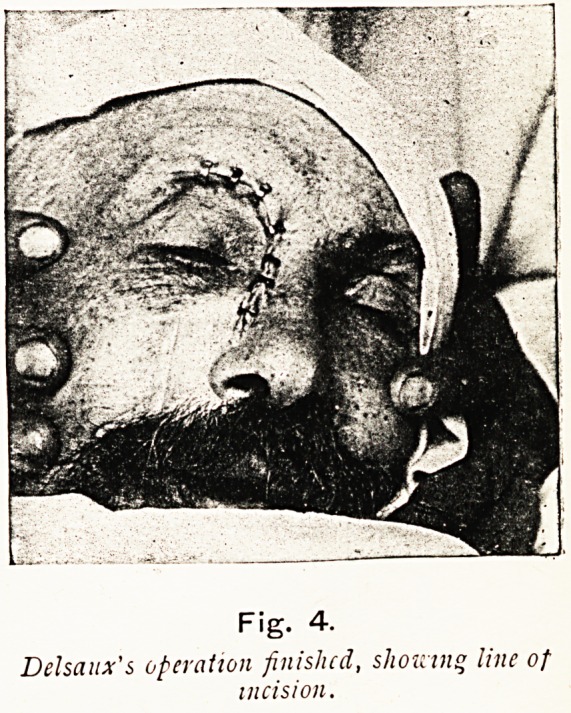


**Fig. 17. f24:**
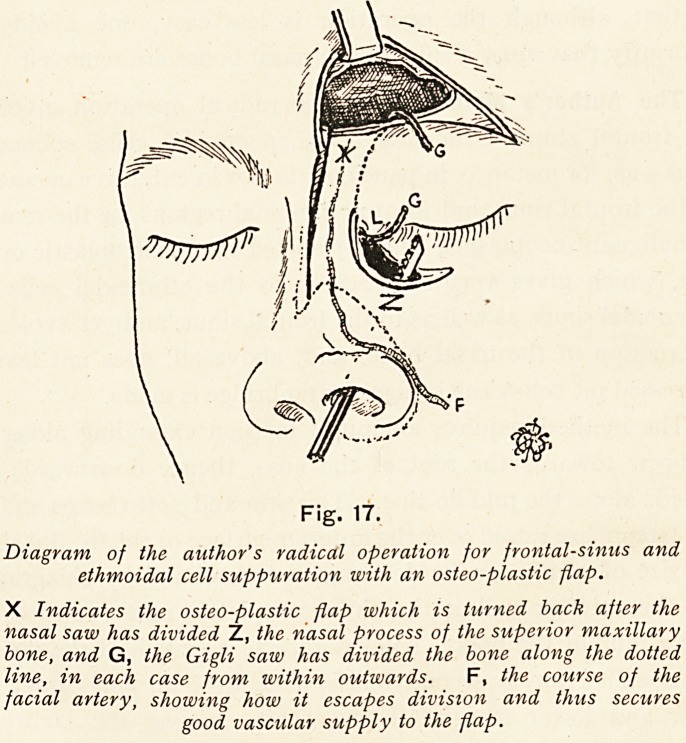


**Fig. 1. f25:**
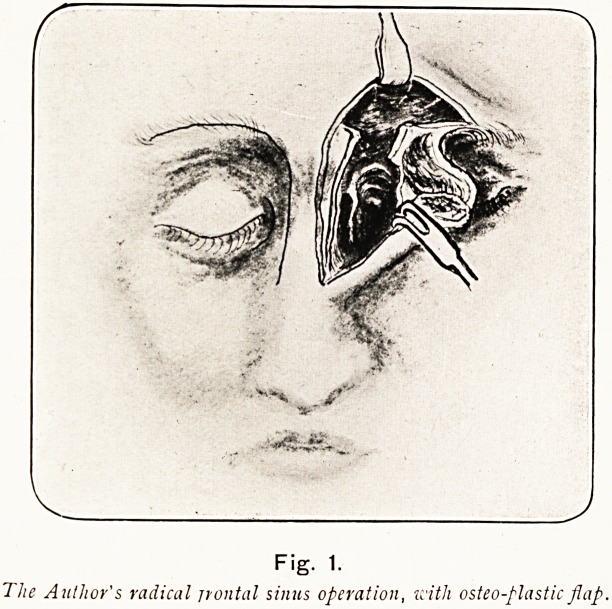


**Fig. 2. f26:**
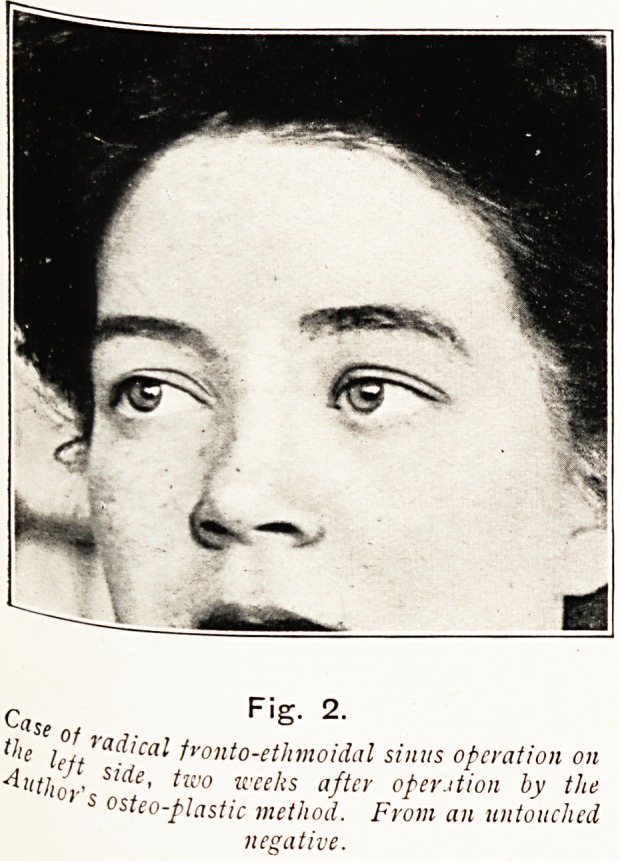


**Fig. 3. f27:**
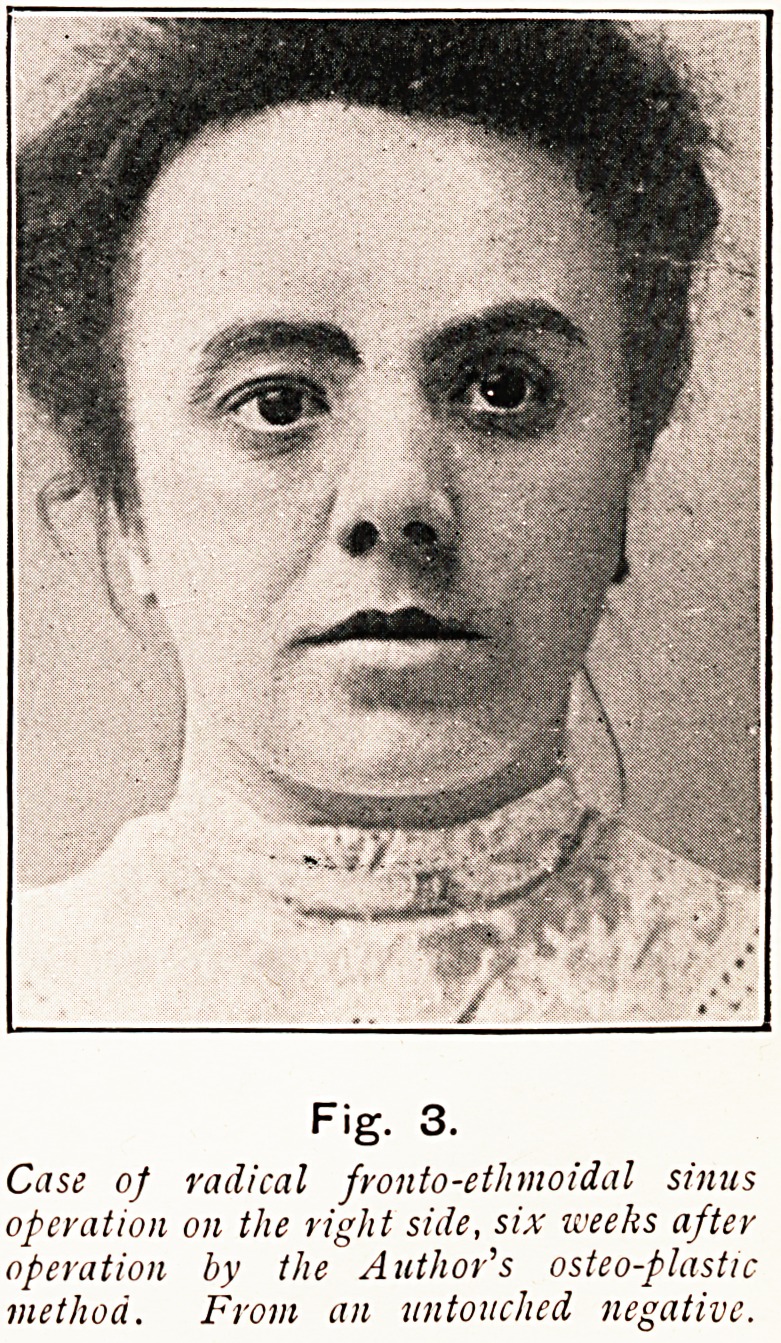


**Figs. 18 and 19. f28:**
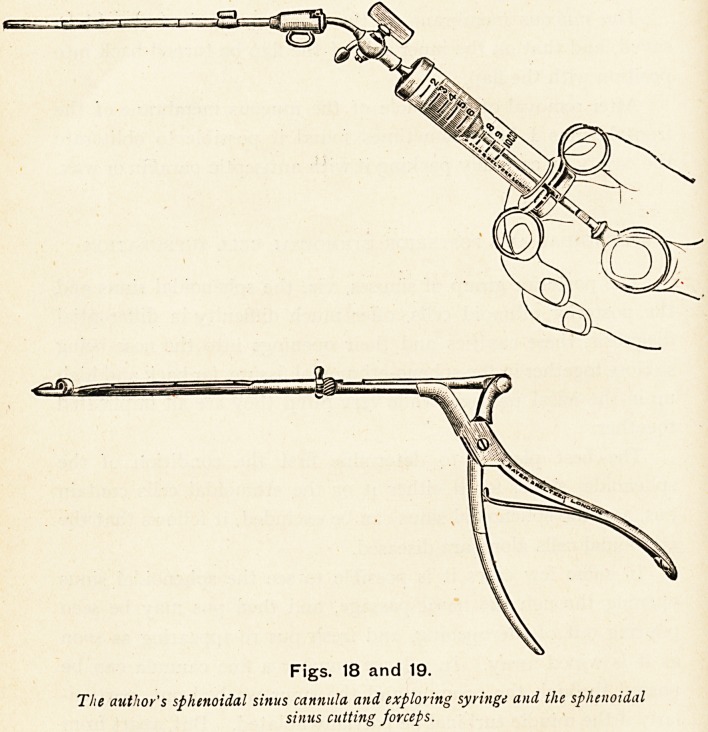


**Fig. 20. f29:**
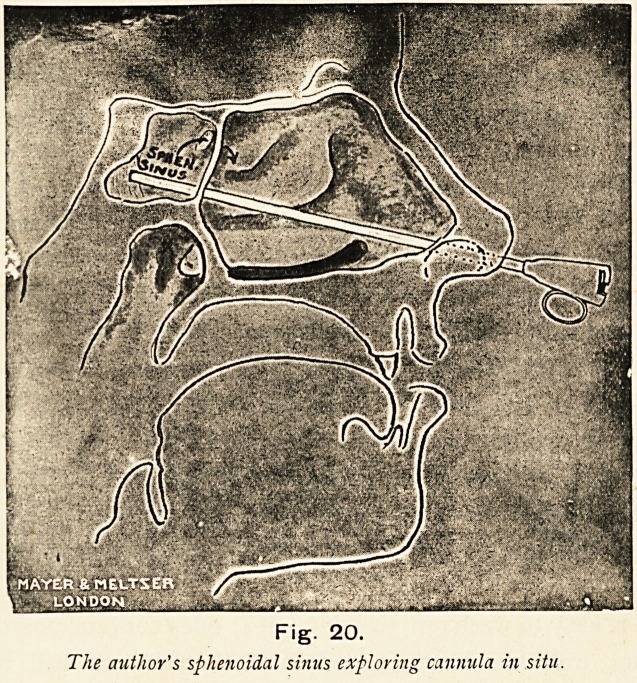


**Fig. 21. f30:**